# Molecular genotyping, diversity studies and high-resolution molecular markers unveiled by microsatellites in *Giardia duodenalis*

**DOI:** 10.1371/journal.pntd.0006928

**Published:** 2018-11-30

**Authors:** Maurício Durigan, Claudio Benício Cardoso-Silva, Maísa Ciampi-Guillardi, Guilherme Toledo-Silva, Gustavo M. Mori, Regina M. B. Franco, Anete P. Souza

**Affiliations:** 1 Centro de Biologia Molecular e Engenharia Genética (CBMEG), Universidade Estadual de Campinas (UNICAMP), Campinas, São Paulo, Brazil; 2 Departamento de Fitopatologia–ESALQ–Universidade de São Paulo, Piracicaba, São Paulo, Brazil; 3 Instituto de Biociências, Campus do Litoral Paulista, Universidade Estadual Paulista (Unesp), São Vicente, Sao Paulo, Brazil; 4 Departamento de Biologia Animal, Instituto de Biologia, Universidade Estadual de Campinas (UNICAMP), Campinas, São Paulo, Brazil; 5 Departamento de Biologia Vegetal, Instituto de Biologia, Universidade Estadual de Campinas (UNICAMP), Campinas, São Paulo, Brazil; Walter and Eliza Hall Institute, AUSTRALIA

## Abstract

**Background:**

*Giardia duodenalis* (synonyms *G*. *lamblia* and *G*. *intestinalis*) is an enteric protozoan parasite of a wide range of mammalian hosts, including humans and various domestic and wild animals. There is considerable genetic variability in *G*. *duodenalis* and isolates of this parasite have been divided into eight genetic assemblages. Microsatellites markers can be used to discriminate isolates with a high level of sensitivity. This study was conducted to identify and characterize genomic microsatellites (simple sequence repeats—SSRs), sequences of one- to six-nucleotide motifs repeated in tandem, present in the available genomes of *G*. *duodenalis* and to develop new markers that can serve as a tool for detection and for characterizing the genetic diversity of this parasite.

**Methodology/ Principal findings:**

For each genetic assemblage, polymorphism levels for the microsatellite markers were evaluated. After performing the analysis using the MISA and SciRoKo software, 1,853 simple sequence repeats (SSRs) were identified. In all the genomes, trinucleotide repeats were the most common class followed by tetranucleotide. Many of the SSR loci are assemblage-specific, and 36 SSR loci shared among all the genomes were identified. Together with hypothetical proteins, variant-specific surface proteins represented nearly half of the annotated SSR loci. The results regarding the most common repeat among the SSRs led us to infer that positive selection occurred to avoid frameshift mutations. Additionally, based on inter- and intra-genetic assemblages polymorphism analyses, we unveiled previously undetected genetic variation, indicating that the microsatellite markers we developed are useful molecular tools for epidemiological inferences based on population genetics patterns and processes.

**Conclusions:**

There is increasing demand for the development of new molecular markers and for the characterization of pathogens at a higher resolution level. In this study, we present 60 *G*. *duodenalis* microsatellites markers that exhibited high polymerase chain reaction (PCR) amplification efficiency among the different genetic assemblages. Twenty of these markers presented nucleotide sequence polymorphisms and may be used as a genotyping tool. The monomorphic markers can be used for the detection of the parasite at the species and genetic assemblage level. These polymorphic markers revealed a genetic diversity that was previously undetectable, thus they can be considered valuable molecular tools for high resolution markers in future studies investigating *Giardia* and may also be used for epidemiological inferences based on populations genetics patterns and processes.

## Author summary

*Giardia duodenalis* is a protozoan parasite that causes giardiasis, a disease that affects millions of people worldwide and a wide range of mammalian hosts. *G*. *duodenalis* presents considerable genetic variability and there is an increasing demand for the development of new molecular markers. This study aimed to identify and characterize genomic microsatellites of *Giardia* based on the different available genomes. After performing an analysis using MISA and SciRoKo software, 1,853 simple sequence repeats (SSRs) were identified and many of them were annotated as variant-specific surface proteins. We detected 20 polymorphic SSRs, including one shared among all available genomes. Because the markers are based on a simple PCR, they have a great potential for use in future studies of *Giardia* through PCR, genetic sequencing or genotyping by real-time PCR.

## Introduction

*Giardia duodenalis* (synonyms *G*. *lamblia* and *G*. *intestinalis*) is an enteric protozoan that parasitizes a wide range of mammalian hosts, including humans and various domestic and wild animals [1,2]. This parasite causes giardiasis, which is one of the most prevalent parasitic waterborne diseases in the world, accounting for approximately 280 million cases annually [3]. According to a recent review that assessed 381 reported outbreaks attributed to waterborne transmission of parasitic protozoa between 2011 and 2016, 37% (142) of the outbreaks were caused by *G*. *duodenalis* [4].

There is considerable genetic variability in *G*. *duodenalis*, and based on genotyping tools, isolates of this parasite have been divided into eight genetic assemblages (A-H), two of which (A and B) exhibit sub-structuring (AI, AII, AIII, BIII and BIV) found in both humans and animals. The other groups (C-H) are considered host-specific in other animals and rarely parasitize humans [5–7].

*G*. *duodenalis* has been considered to be a single species based on the morphological similarity among isolates. However, based on the extensive genetic variation among different isolates, this parasite can be considered a species complex [8,9]. Evidence supporting this hypothesis is based on the genomic differences among the isolates that have been sequenced [10,11] and comparisons of the divergence among different species of protozoan pathogens [12], which has led to the assignment of species names according to host specificity [13].

Genotyping tools based on the *tpi*, *gdh*, and *bg* genes have been historically used for the identification of genotypes of *G*. *duodenalis* using approaches such as restriction fragment length polymorphism (RFLP), polymerase chain reaction (PCR), real-time pCR (qPCR), and DNA sequencing [2,6,14]. Although multilocus sequence typing tools (MLST) have been leading current efforts for subtyping genetic assemblages according to the presence of unique single nucleotide polymorphisms (SNPs), there are currently no high-resolution MLST tools for *G*. *duodenalis* [7]

Microsatellites (simple sequence repeats—SSRs) are sequences of one- to six-nucleotide motifs repeated in tandem. These sequences are highly polymorphic, multi-allelic markers that present codominant Mendelian inheritance and are considered ideal markers for population and diversity studies [15]. These markers can be used to discriminate isolates with a high level of sensitivity [16], depending on the population structure of the parasite, the level of genetic exchange and the number of markers used [17].

The use of high-resolution markers allows for the identification of many genotypes that cannot be detected using conventional markers. In addition to genotyping parasites into the pre-existing subtypes, such identification allows for the characterization of individuals at a higher level of resolution, which can be exploited to investigate the epidemiology of disease and the role of asymptomatic carriers, as well as to track the disease source through contaminated water and food [18].

There are significant differences among the available genomes in nucleotide sequences, gene repertoire, and surface molecules [7,12,19,20]. The wide variation in these classic molecular markers among the different genetic assemblages suggests that these could be different species [12]. The development of new MLST tools is needed for high-resolution typing and population genetic characterization of *G*. *duodenalis* [7]. This study was conducted to identify and characterize the genomic microsatellites present in different genetic assemblages of *G*. *duodenalis* as potential candidates for new polymorphic markers that can be used for the detection of the parasite and may also contribute for population genetics-based epidemiological studies.

## Methods

### Identification of microsatellites in the WB (AI), DH (AII), GS (BIV) and P15 (E) genomes

**Fig 1:**
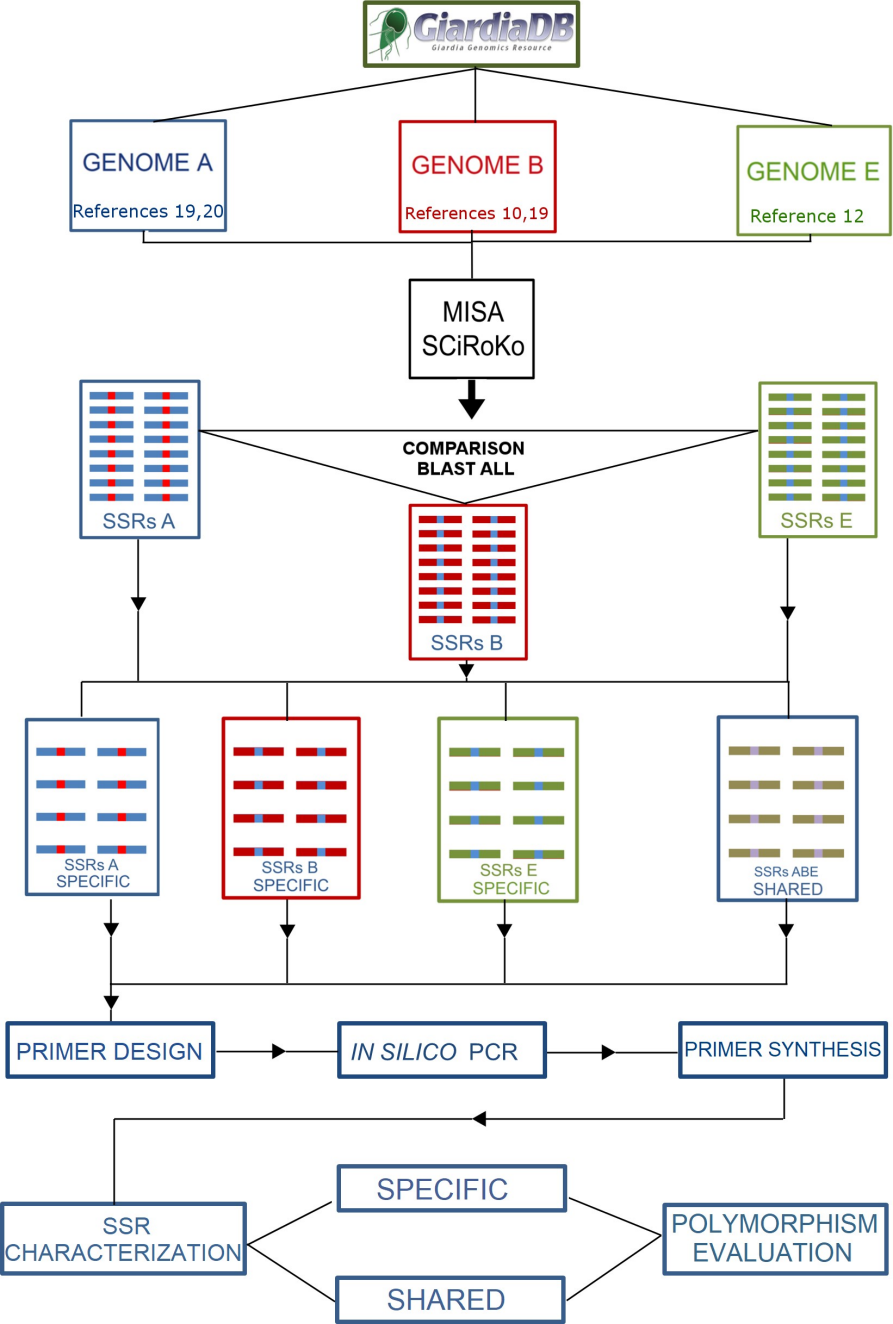
Flowchart of the methodology used to characterize the microsatellites in *G*. *duodenalis*.

The complete published *G*. *duodenalis* genome sequences [10,12,19–21] were obtained from GiardiaDB v5.0 [22]. The identification of putative microsatellites in each genome was performed using the software packages MISA–Microsatellite Identification Tool [23] and SciRoKo [24], which have both been previously validated for polyploid organisms [25,26]. The criteria for the SSR identification included sequences that presented at least five dinucleotide repeats, four trinucleotide repeats, or three tetra-, penta-, or hexanucleotide repeats. After identifying SSRs, the adjacent 300-bp regions downstream and upstream of each SSR were determined as the microsatellite locus. An overview of the methodology is presented as a flowchart in Fig 1.

### *In silico* analysis of transferability

All available genomes corresponding to different genetic assemblages were used to evaluate the conservation and uniqueness of the microsatellite loci including their adjacent upstream and downstream regions. A customized script was used to edit the genomes and generate all loci sequences (contigs); these sequences were then compared to each other using BLASTn [27] software with parameters that were personalized to recognize regions with high similarity (e-value ≤ 1e-100). The analyzed loci were classified as specific for one genome or shared between two or more genomes. The number of SSR loci analyzed was slightly lower than the total number of identified SSRs because some microsatellites were present at the very beginning or end of some contigs, which prevented primer design and further analyses. To remove possible redundancies and duplicated loci, each sequence containing an SSR was aligned against the same genome in which it had been identified. The loci that were present in multiples copies along the genome were excluded from the analysis.

### Functional annotation of SSR loci

The microsatellite sequences were searched against the nr protein database (National Center for Biotechnology Information–NCBI) using BLASTx [28] with an e-value cut-off of 1e-5. Gene ontology (GO) terms were obtained using the default parameters for GO mapping with the Blast2GO software [29] and the annotation procedure used a cut-off e-value of 1e-10 [30]. First-level GO annotations were removed because these annotations were not informative. To identify possible differences exclusive to the different genetic assemblages, a GO enrichment analysis was performed using Fisher’s Exact Test considering results with a false discovery rate (FDR) < 0.05. Annotated sequences were matched against the Pfam [31] database to identify protein domains using the InterProScan tool from Blast2GO. SSRs sequences that corresponded only to a very small fragment of a gene were excluded from the functional analysis.

### Primer design and characterization of microsatellites

Complementary primers were designed for the conserved regions adjacent to the microsatellites using the Primer 3 Plus [32] and Primer Select programs (DNAStar, Madison). SSR loci were chosen based on different motifs, repeat types and numbers of SSR repeats. Some primers were designed to prime intergenic regions and others at coding regions of important *Giardia* proteins such as kinases, ankyrin, variant-specific surface proteins (VSPs) and hypothetical proteins.

Primers were designed to detect specific genetic assemblages and SSR loci shared among all *Giardia* available genomes. The primers were first tested using the software FastPCR [33], which performed *in silico* PCR assays against the genome database. The comprehensive list of primer sequences of microsatellite loci used in this study and their corresponding positions in the contigs are presented in S1, S2, S3 and S4 Tables.

DNA samples from specimens from different genotypes such as AI, AII, BIII, BIV, C, D, and E used in this study were evaluated in previous studies to confirm their corresponding genetic assemblages and the absence of mixed assemblages [34,35]. Only samples undoubtedly assigned to a specific genetic assemblage and with no evidence of mixed assemblages according to conventional markers such the *tpi*, *gdh* and *bg* genes, were used in this study. After this selection it was included in this study 23 isolates from genetic assemblage A (22AII and 1 AI), 23 isolates from genetic assemblage B (18 BIV and 5 BIII), 5 isolates form genetic assemblage C, 5 isolates from genetic assemblage D and one isolate from genetic assemblage E. A complete list with all isolates used in this study including the corresponding genetic assemblages and their source is provided in S5 Table.

Primer pairs were evaluated using a touchdown PCR assay [36]. The thermocycler parameters were as follows: 94°C for 2 min; 10 cycles of 94°C for 1 min, 65°C (-1°C/cycle) for 1 min and 72°C for 2 min; 18 cycles of 94°C for 1 min, 55°C for 1 min and 72°C for 2 min; and 72°C for 5 min. The PCR products were visualized on TBE 0.5x 3% agarose gels stained with ethidium bromide followed by vertical electrophoresis in TBE 1x 6% polyacrylamide gels that were silver stained [37]. Specific protocols used in our study were deposited in protocols.io under the access numbers dx.doi.org/10.17504/protocols.io.nyzdfx6 for the touchdown PCR and dx.doi.org/10.17504/protocols.io.nz3df8n for the preparation of polyacrylamide gels and silver staining.

After the alignment analysis, the microsatellites and adjacent regions that presented high similarity to several regions of the same genome were eliminated from further analyses. The same procedure was followed for the primers that presented unspecific amplification or no amplification in the desired genomes.

### Polymorphism analysis and transferability between genetic assemblages

All SSR loci that were considered suitable in the specificity test, were characterized in the polymorphism analysis. Polymorphism levels for the microsatellite markers were evaluated for each genetic assemblage. Because of the polyploid nature of the organism, microsatellite data were converted to a binary format as dominant data. The product sizes were determined by comparison with a 10bp DNA ladder (Invitrogen, Carlsbad, CA). The same specimens for each genetic assemblage were used to identify polymorphisms of the genetic markers. The primers designed for the genetic assemblage E could not be included in the polymorphism analysis because, apart from the specificity test in the first analysis, there was no set of samples from this genetic group to define a population to be tested. The allelic polymorphic information content of each SSR was calculated using the polymorphism content index (PIC) = $1-\sum_{i=1}^{n}p_{i}^{2}-\sum_{i=1}^{n}\sum_{j=i+1}^{n}2p_{i}^{2}p_{j}^{2}$, where n is the number of alleles of the marker among the set of genotypes used for characterizing the SSR polymorphism, and p_i_ and p_j_ are the frequencies of alleles i and j. The observed and expected heterozygosity were calculated using the software TFPGA [38].

To screen for genetic variation at inter- and intra-assemblage levels, thus validate the application of the microsatellite markers developed in this study as tools population genetics-based epidemiological studies, the genotyping profiles obtained in this study were compared with the conventional *Giardia* molecular markers (gdh, tpi and bg) as the DNA samples used in this study were previously analyzed [34,35]. Additionally, we analyzed one dataset composed only by isolates from genetic assemblages AI and AII and another composed by samples from genetic assemblage BIV. The former was genotyped using 17 polymorphic microsatellites characterized in this study whereas the latter was genotyped using 11 loci and these datasets were considered separately in further analyses. We identified unique multilocus genotypes (MLG), a proxy for detecting clones using poppr 2.6.1 [39] implemented in ade4 1.7–10 [40] and adegenet 2.1.1 [41].

### Ethics statement

No specific permissions were required for the environmental locations where the samples were collected. This study did not involve endangered or protected species. The clinical samples were collected as part of another study (https://doi.org/10.1371/journal.pone.0115489) and ethical approval was granted by the ethic committee of the Faculdade de Ciencias Medicas Unicamp (251–2009).

## Results

### Identification of the SSR marker in the WB_AI, DH_AII, GS_B and P15_E genomes

After the analyses were performed using the MISA and SciRoKo software, a total of 1,853 SSRs were identified, including 506 SSRs in the WB_AI genome, 438 SSRs in the DH_AII genome, 402 SSRs in the GS_B genome and 507 SSRs in the P15_E genome. The total number of SSRs according to each type of repeat is presented in Table 1.

**Table 1:** Number of microsatellites detected in the different genomes.

Repetition/Genome	AI [20]	AII [19]	B [10]	E [12]
**Di-5**	78	77	131	84
**Tri-4**	206	156	123	207
**Tetra-3**	163	150	120	158
**Penta-4**	21	21	18	32
**Hexa-3**	38	34	10	26
**Total**	506	438	402	507

This table presents the number of microsatellites in the different genomes of *G*. *duodenalis* according to the different repetition types.

**Fig 2:**
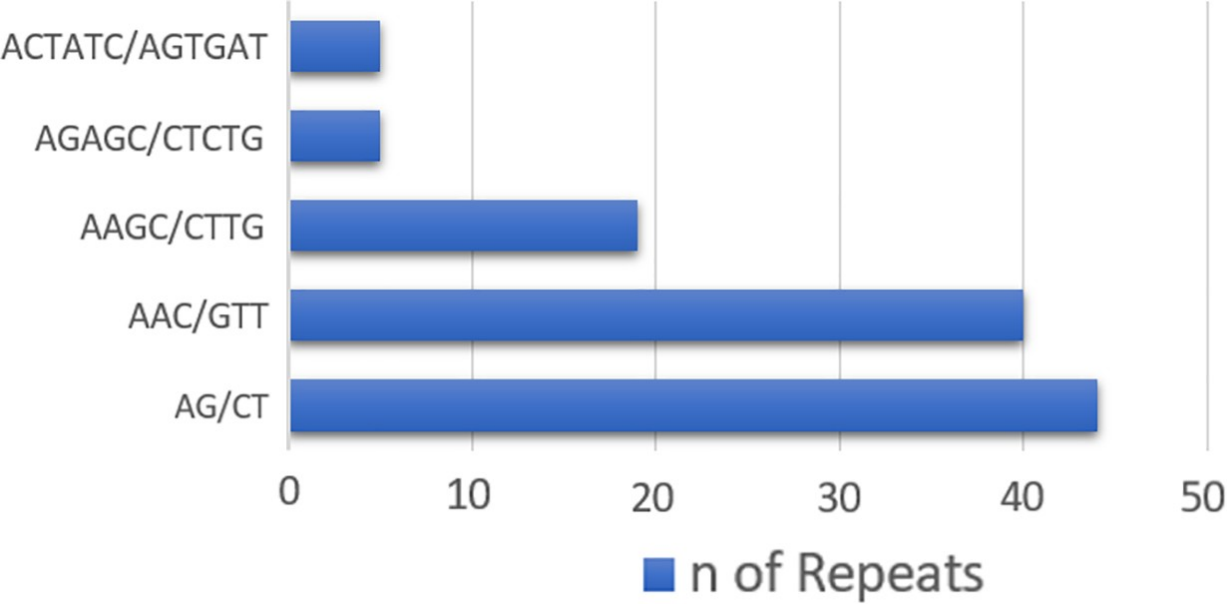
Frequency of classified repeat types. Frequency of the most common motifs according to each repeat type (considering the complimentary sequence).

Some SSR repeats and motifs were identified at higher frequencies than others. Trinucleotide repeats were the most common class in all the genomes, followed by tetranucleotide repeats. According to the frequency analyses, the di-, tri-, tetra-, penta-, and hexanucleotides were most commonly present with five, four, three, three and three repeats, respectively, in all genomes. Fig 2 presents the frequency of the most frequently identified SSR motifs (considering sequence complementary) for all the different types of repeat classes.

### *In silico* analysis of transferability among identified SSRs in the available genomes

Analysis of the microsatellites present in the available genomes allowed us to identify SSRs and their adjacent regions shared among all genomes and specific SSRs present in only one genome. We also identified some SSR loci present in more than one but not all genomes.

**Fig 3:**
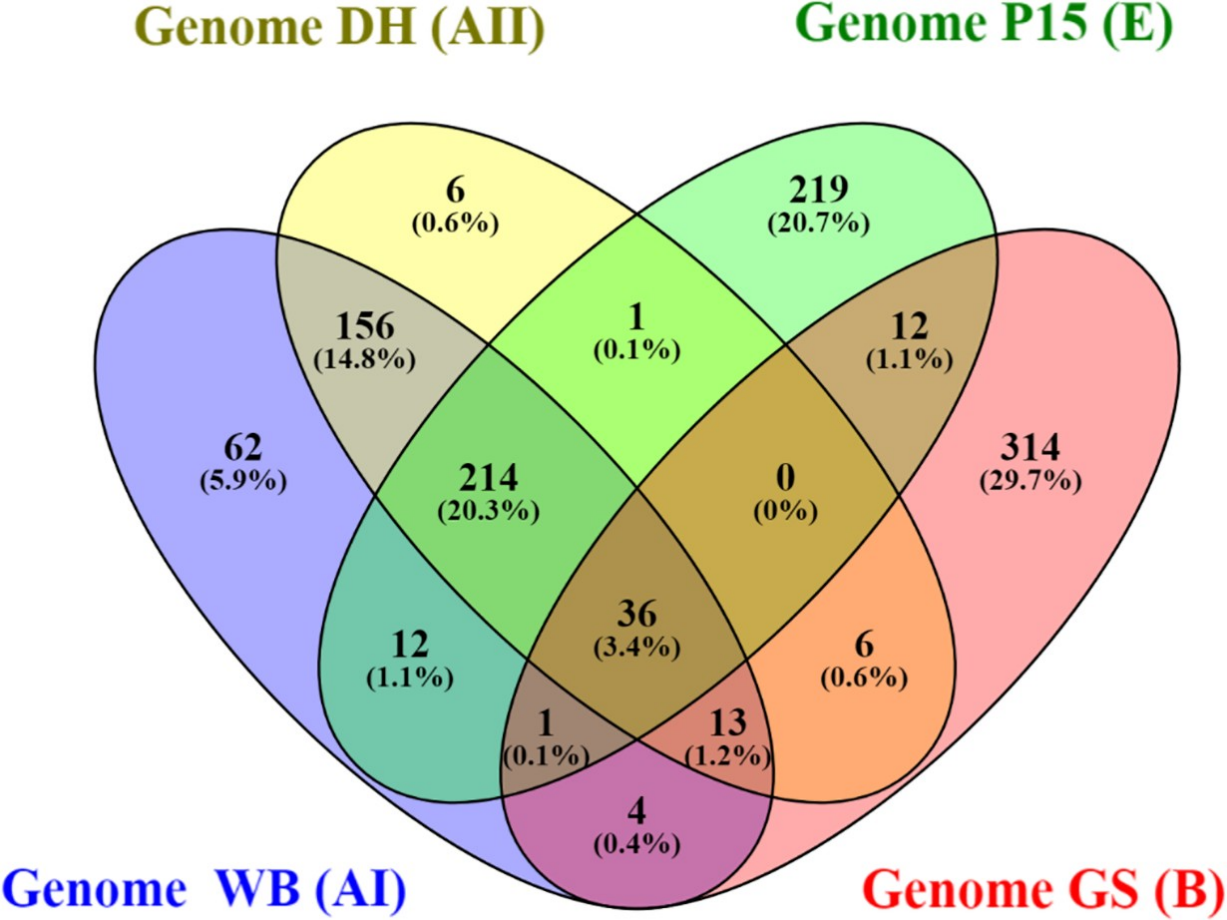
*In silico* transferability of *G*. *duodenalis* SSRs among the different genomes.

The *in silico* analysis of transferability across the different genetic assemblages showed that many of the SSR loci were assemblage-specific, although most of the SSR loci of genetic assemblages AI and AII were shared between these two genomes. Only a small percentage of SSRs appeared to be shared among all different genomes. The results regarding the transferability of the SSR loci in each genome are presented as a Venn's diagram [42] (Fig 3).

After searching for SSRs present in all available *Giardia* genomes, 36 SSR loci were identified. Most of these SSRs were trinucleotide repeats, and the most common repeat (after normalization) was (CTT)_4_.

### Functional annotation

**Fig 4:**
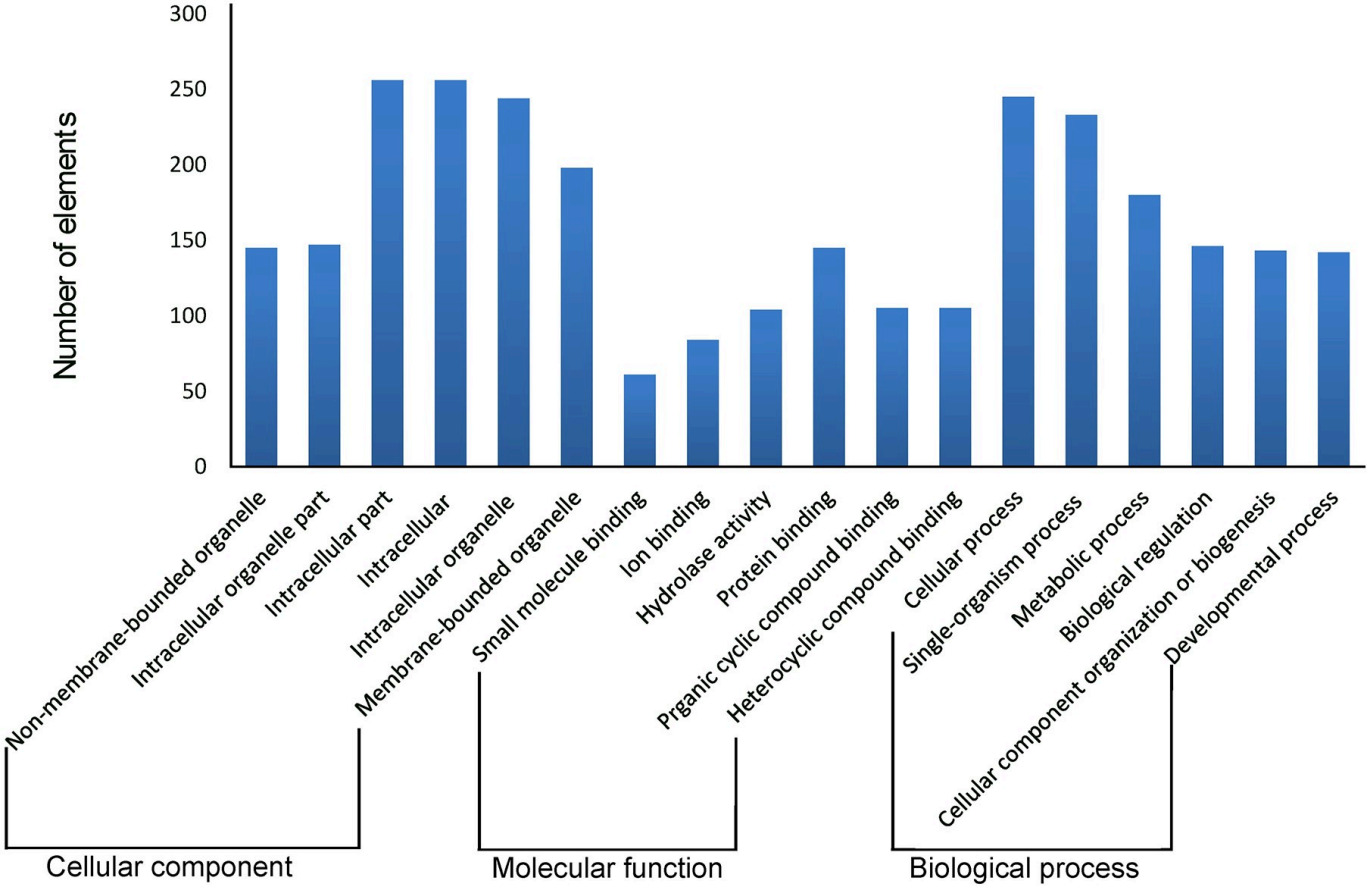
Gene ontology classification of *G*. *duodenalis* genes. Distribution of the GO categories assigned to the *G*. *duodenalis* genomes. Genes were classified into three categories: cellular components (Level 3), molecular functions (Level 3) and biological processes (Level 2). Regarding the GO annotation, 921 (50%) SSR loci were validated by the Blast2GO annotation procedure, resulting in 2,793 GO terms: cellular component terms composed most of the functional vocabulary (33.90%), followed by the molecular function (33.62%) and biological process (32.47%) classes. Within the biological process category, metabolic processes (99), protein phosphorylation (87) and the serine family amino acid metabolic process (71) figured prominently. Regarding the molecular function category, ATP binding (181) and protein serine/threonine kinase activity (66) were the most represented GO terms. Half of the cellular component class of annotations was related to integral components of the membrane (465).

Of the 1,842 SSR loci that could be analyzed, 1,567 (85.07%) were matched to a protein in the NCBI nr database. Of the matched proteins, 578 (36.88%) were classified as hypothetical proteins and 206 (13.15%) were classified as VSPs; together these represented nearly half of all the annotated SSR loci. The different genomes presented similar distributions of blast hits, except for isolate AII, which exhibited lower rates of VSPs than the other assemblages. Fig 4 presents the top six GO results based on the cellular components, molecular function and biological process categories, respectively. A comprehensive list of all GO results is presented in S6, S7 and S8 Tables. GO enrichment analysis showed that no particular GO term was enriched (FDR < 0.05) when conducting pairwise comparisons of all the assemblages.

The most significant functional profile for the SSR loci arose from kinases, which are involved in the phosphorylation of many proteins [20]. This result was reflected in the main GO terms detected in the classes of molecular function (ATP binding [GO:0005524] with 181 terms and protein serine/threonine kinase activity [GO:0004674] with 66 terms) and biological process (protein phosphorylation [GO:0006468] with 87 terms and the serine family amino acid metabolic process [GO:0009069] with 71 terms). A comparison of the SSR sequences against the Pfam domain database resulted in 160 loci matching at least one protein domain model. Considering the different genetic assemblages, the most common families among the annotated proteins in all cases were *Giardia* VSPs (PF03302, 82 loci) and ankyrin repeat-containing domain (PF12796, 18 loci).

### PCR amplification and characterization of microsatellites

Considering all the available genomes, 1,853 microsatellites were identified by the *in silico* analysis. A total of 20 primer pairs specific for genetic assemblage B and 20 specific for genetic assemblage E were designed. Regarding genetic assemblage A, 40 primer pairs were designed to prime both genomes AI and AII (genetic assemblages commonly associated with human samples and other animals), generating a total of 80 SSR loci for characterization and specificity analysis.

Regarding genetic assemblage A, 28 primer pairs specifically amplified only samples from this genetic assemblage. For genetic assemblage B, 11 primer pairs produced specific amplification products, and 17 primer pairs were specific to genetic assemblage E. A total of 56 primer pairs produced coherent amplification products. Primer pairs that did not result in any amplification products or produced nonspecific fragments were not further analyzed. The same procedure was performed with primer pairs that generated amplification products from unexpected genetic assemblages.

We also aimed to identify SSR loci shared between all available genomes that could provide genetic markers for the detection of the parasite. Primer pairs that did not result in any amplification products or did not generate fragments in all the specimens tested were not further analyzed. Thirty-six primer pairs were designed for the 36 loci present in all the available genomes. A total of four primer pairs generated coherent amplification products in all the tested genetic assemblages. The results of the amplification reactions are shown in S9, S10, S11 and S12 Tables.

### Polymorphism analysis

Results regarding the characterization of SSR polymorphic loci in specific genetic assemblages and shared loci are provided in Table 2. Regarding genetic assemblage A, in which 28 loci produced coherent amplification products, 12 loci were polymorphic and the remaining 16 loci were considered monomorphic (S13 Table). Considering the polymorphic markers, the mean number of alleles was 2.25 (2–4). The mean H_E_, H_O_ and PIC values were 0.241 (0.085–0.507), 0.037 (0.00–0.181) and 0.202 (0.079–0.538), respectively. GduA24 presented the highest number of alleles and the highest PIC value.

**Table 2:** Characterization of SSR loci in specific genetic assemblages and shared loci.

Locus	Number of alleles	Allelic variation (bp)	H_E_	H_O_	PIC
GduA01	2	224–228	0.1765	0	0.1574
GduA02	2	232–234	0.1287	0	0.1167
GduA08	2	139–141	0.085	0.087	0.0797
GduA10	2	168–170	0.5077	0	0.3724
GduA16	2	168–170	0.4965	0.0833	0.3679
GduA17	2	246–249	0.0888	0	0.083
GduA19	2	242–251	0.085	0	0.0797
GduA20	2	217–220	0.1765	0.0952	0.1574
GduA21	3	218–242	0.2718	0	0.2468
GduA24	4	175–187	0.63	0.1818	0.5388
GduA25	2	189–198	0.0929	0	0.0865
GduA26	2	248–251	0.1594	0	0.141
GduB01	5	209–230	0.4508	0.1111	0.3962
GduB02	3	153–165	0.591	0.1	0.5108
GduB03	3	173–185	0.2079	0.1111	0.1899
GduB05	3	216–234	0.3763	0	0.3257
GduB06	5	188–224	0.4705	0.0435	0.4342
GduB09	2	250–253	0.241	0	0.2078
GduB10	2	244–250	0.2319	0	0.201
GduABE01	2	330–333	0.043	0	0.0415

This table presents the results of each SSR polymorphic that were considered suitable for genotyping analysis. HE means expected heterozygosity Ho means observed heterozygosity PIC means polymorphic information content. The allelic variation represents the length variation of the alleles in the population. The number of alleles represents the number of alleles in the population.

Among the 11 loci of genetic assemblage B that were successfully amplified in the previous assays, seven loci were polymorphic, and the four remaining loci were considered monomorphic (S14 Table). For the polymorphic markers, the mean number of alleles was 3.28 (2–5). The mean H_E_, H_O_ and PIC values were 0.367 (0.207–0.591), 0.052 (0.00–0.111) and 0.323 (0.18–0.509), respectively. GduB01 and GduB06 presented the highest number of alleles, and GduB02 presented the highest PIC value.

Among the four loci shared among all the tested genetic assemblages, GduABE01 was polymorphic. For this locus, the H_E_, H_O_ and PIC values were 0.043, 0.00 and 0.0415, respectively. All three remaining markers were monomorphic (S15 Table). A figure reporting some of the polyacrylamide gels is provided in S1 Fig. S16 Table is a list of accession numbers/ID numbers for proteins mentioned in the text.

**Fig 5:**
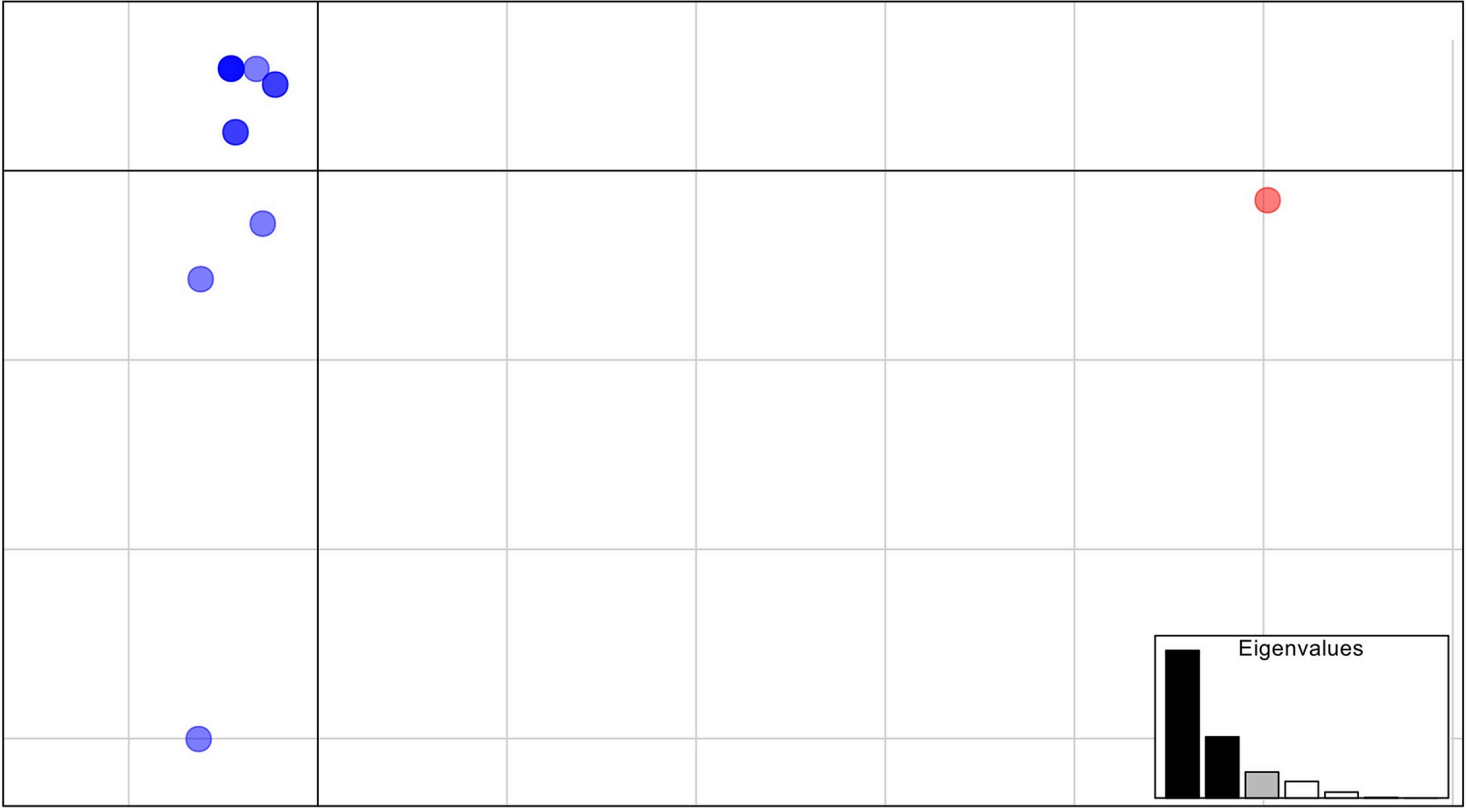
Principal component analysis (PCA) of *Giardia duodenalis* isolates. The isolates had been previously assigned to genetic assemblage BIV according to their multilocus genotypes based on tpi, gdh, and bg genes. Red and blue denote for two separated intra-assemblage clusters.

As the DNA samples used in this study were the same ones from our previous studies [34,35] we could compare some of the SSR results with the conventional *Giardia* molecular markers (gdh, tpi and bg) regarding the molecular genotyping of the samples. SSR profiles of the polymorphic markers GduA10, GduA16, GduA20, GduA24 showed that samples HC31, HC48 and HC22, considered clonal according to the conventional (MLG BRA01) markers [34] were different based on the MLG profiles obtained by microsatellites we characterized. Using these high-resolution markers allowed us to detect 12 and 17 unique MLG (clones) out of the 13 and 19 samples for the AI and AIII dataset and B dataset. Additionally, we observed that A assemblage samples were consistently separated by PCA in two clearly separated clusters, corresponding to samples from AI and AII assemblages (S2 Fig). In this analysis, the first and second Principal Components (PCs) retained 57.31% and 23.74% of total variance. Regarding B assemblage, PCA, whose first PCs accounted for 30.09% and 21.76% of the observed genetic variability, revealed that samples were organized into two clear intra-assemblage clusters (Fig 5).

## Discussion

In this study, we present 60 new *G*. *duodenalis* microsatellite markers that exhibited high PCR amplification efficiency and can be used for detection of the parasite at the species and genetic assemblage level. Of these, 20 markers presented nucleotide sequence polymorphism and may also contribute for diversity studies. Microsatellites have been observed in animals [43], [44], plants [45], [46] [47], fungi [48] and protozoa, [49] [50]. [51]. [52,53] and these studies have been successful in characterizing the genetic diversity of these organisms. The genome of *G*. *duodenalis* presents a high proportion of genes with little non-coding regions, and most of the microsatellites identified in this study were based on trinucleotide repeats. Most tetranucleotide repeats (the second most common type of repeat in this study) in the genome of *G*. *duodenalis* consisted of multiple three repeat units. This result led us to infer that positive selection has occurred to avoid frameshift mutations.

Notably, only a few of the detected microsatellite loci (36 of 1853) are shared among all available *G*. *duodenalis* genomes. The presence of the SSR loci in the different genomes suggests highly conserved regions and, therefore, these experimentally validated genetic markers can be used for the detection of the parasite. Moreover, for the shared microsatellites that were identified, the PIC values were zero or extremely low, which is also in accordance with the low variability of these loci. The comparisons made among the microsatellite loci developed in the present study indicated a greater extent of shared SSRs between genetic assemblages AI, AII and E, while simultaneously, both assemblages exhibited great differences from genetic assemblage B. These results corroborate findings obtained by constructing phylogenies of conserved genes; this revealed greater genetic similarity between genetic assemblages A and E than between either of these assemblages and genetic assemblage B [54].

Regarding the functional annotation, at first, it is noted that a majority of the SSR loci were located within or close to coding regions (85.07%). This result is in accordance with the few and sparse intergenic regions reported in *Giardia* genomes [19]. When different isolates were compared, AII presented the lowest rate of no hits in the blast searches. The finding of a higher prevalence of SSR loci that were related to hypothetical proteins, VSPs, ankyrin repeats, kinases and high cysteine membrane proteins (HCMP), can be explained simply by the representativeness of these proteins in the genomes of *Giardia* assemblages [10].

Polymorphisms were identified in several SSR loci that encode important proteins, such as kinases (different types), coiled-coil proteins, IF-1, HSP-90, and many hypothetical proteins (S9, S10, S11 and S12 Tables). We also detected polymorphism of VSP SSRs. This protein is already known to be essential for the development of immunity, immune selection and immune evasion in *Giardia*. Each organism expresses only one VSP at a time, and RNAi is likely the mechanism underlying post-transcriptional regulation of VSP transcripts because all members of the RNAi machinery are present in the *Giardia* genomes [55]. SSRs were located near VSPs, and this positioning could be a hypothetical mechanism for increasing antigenic variation, adding to the already unique VSP diversity among isolates [56]. The Pfam domain *Giardia* variant-specific surface protein (PF03302) was the most prevalent among the analyzed SSR loci, with a higher representation in isolate E.

The SSR primers designed for the polymorphic loci of the genetic assemblage A exhibited potential applicability for further analyses of the intraspecific genetic diversity in both genomes (WB and DH). The results we obtained showed the differences among isolates that had been considered clonal by their unique MLG based on conventional markers [34,35], namely HC31, HC48, HC22. This finding highlights previously unveiled genetic variability of *G*. *duodenalis* samples, attesting usefulness of the microsatellite described in this study as a complementary method to characterize the genetic diversity of a *Giardia* populations. Additionally, the population genetic structure we observed in PCA analyses support these results as it is possible to clearly tell apart isolates from different assemblages (AI and AII, S2 Fig) and isolates from the same genetic assemblage (BIV). Together, these results demonstrate how the microsatellite markers characterized herein are valuable tools for epidemiological studies supported by population genetics inferences regarding *G*. *duodenalis*.

The data in this study reveal a highly distinct pattern of variation among the markers developed for genetic assemblages A and B, in both the number of alleles found and the polymorphism information content in the loci. Differences in the frequency of heterozygosity among these genetic assemblages, previously described in other studies [10,34], were verified using the identified microsatellite loci and corroborate the species complex hypothesis in this organism.

Many SSR loci were detected in more than one region of the same genome. This phenomenon prevented the use of these loci in designing primers. Microsatellites frequently may be associated with repetitive regions, such as transposable elements or proteins of antigenic variation. The association between microsatellites and repetitive elements has been previously documented in vertebrates and plants. Dinucleotide motifs, especially CA/GT, are commonly associated with repetitive elements [57].

In this study, we detected 20 new markers which were genotyped as polymorphic in the different genetic assemblage. The sequencing of certain *G*. *duodenalis* genomes presented low levels of allelic heterozygosity, which was particularly evident in the WB_A genome. Although higher in the GS_B genome, this rate/level may still be considered low. One hypothesis is that a heterozygous individual may have variability within the nucleus and that such variation is expressed through different alleles. We also cannot exclude the possibility that variation between different nuclei occurred through genetic exchange or lateral gene transfer [12]. The lack of heterozygotes in *G*. *duodenalis* has been considered an indicator of the clonal evolution of the parasite through purifying selection and gene conversion, whereas an excess of heterozygotes is considered evidence of hybridization or recent sexual events [58].

Most of the tested SSR loci were monomorphic according to the tested population. Considering that *Giardia* genomes are small and highly compact [20], this result may be a consequence of the high frequency of conserved genes across all genomes, which prevents genetic diversity in these loci. Regarding the shared SSR loci, one molecular marker was detected as polymorphic across all tested genetic assemblages. Shared loci are expected to be conserved, which significantly reduces the probability of polymorphism. The GduABE01 locus is promising for future studies that may evaluate the levels of GduABE01 polymorphism by genotyping a wide range of individuals and by sequencing of the entire locus.

### Conclusion

In this study, we reported the development of microsatellite markers for *G*. *duodenalis*. The developed primers (including the specific monomorphic markers) can be used for detection of *G*. *duodenalis* in clinical and environmental samples and to identify the major genetic assemblages of the species. Because the markers are based on a simple PCR, these primers may represent an accessible method for diagnostic sanitation companies, diagnostic institutions (including zoonosis control centers, veterinary clinics and diagnostic laboratories) and research institutions.

The increasing demand for the development of new informative loci will define whether the new molecular markers made available in this research will be used for assessing allelic polymorphisms through PCR, genetic sequencing or genotyping by real-time PCR. Furthermore, the molecular markers developed in this study presented a considerable diversity in alleles and high PIC values. These polymorphic markers revealed a genetic diversity that was previously undetectable [34,35], thus they can be considered valuable molecular tools for high resolution markers in future studies investigating *Giardia* and may also be used for epidemiological inferences based on populations genetics patterns and processes.

## Supporting information

S1 Table Designed primers, fragment size, SSR motif and position in the *Giardia* assemblage A (WB) genome. (DOCX)

S2 Table Designed primers, fragment size, SSR motif and position in the *Giardia* GS genome. (DOCX)

S3 Table Designed primers, fragment size, SSR motif and position in the *Giardia* assemblage E genome. (DOCX)

S4 Table Designed primers, fragment size, SSR motif and position of *Giardia* primers shared by all assemblage genomes. (DOCX)

S5 Table Specimens included in the present study according to their genotype and source. (DOCX)

S6 Table Gene ontology classification of *G*. *duodenalis* genes for the GO category of Biological process. (DOCX)

S7 Table Gene ontology classification of *G*. *duodenalis* genes for the GO category of Cellular components. (DOCX)

S8 Table Gene ontology classification of *G*. *duodenalis* genes for the GO category of Molecular function. (DOCX)

S9 Table Amplification results and proteins associated with SSR loci in genetic assemblage A. (DOCX)

S10 Table Amplification results and proteins associated with SSR loci in genetic assemblage B. (DOCX)

S11 Table Amplification results and proteins associated with SSR loci in genetic assemblage E. (DOCX)

S12 Table Amplification results and proteins associated with shared SSR loci. (DOCX)

S13 Table Characterization of SSR loci in genetic assemblage A. This table presents the results of each SSR loci in genetic assemblage A that were considered suitable for genotyping analysis. HE means expected heterozygosity Ho means observed heterozygosity PIC means polymorphic information content. The allelic variation represents the length variation of the alleles in the population. The number of alleles represents the number of alleles in the population.
(DOCX)

S14 Table Characterization of SSR loci in genetic assemblage B. This table presents the results of each SSR loci in genetic assemblage B that were considered suitable for genotyping analysis. HE means expected heterozygosity Ho means observed heterozygosity PIC means polymorphic information content. The allelic variation represents the length variation of the alleles in the population. The number of alleles represents the number of alleles in the population.
(DOCX)

S15 Table Characterization of shared SSR loci. This table presents the results of each SSR loci in the shared SSR loci that were considered suitable for genotyping analysis. HE means expected heterozygosity Ho means observed heterozygosity PIC means polymorphic information content. The allelic variation represents the length variation of the alleles in the population. The number of alleles represents the number of alleles in the population.
(DOCX)

S16 Table List of accession numbers/ID numbers for proteins mentioned in the text. (DOCX)

S1 Fig A 6% denaturing silver-stained polyacrylamide gels for the characterization of the GduA17 and GduA20 markers. A—PCR products for the monomorphic marker GduA17 (205bp). B–PCR products for the polymorphic marker GduA20 (242bp) which three different alleles could be identified in the specimens from the same genetic assemblage. The numbering follows the number of the samples in the S5 Table. PC means positive control. NC means negative control. Double bands present in the same individual in this gel are a PCR amplification artifact that normally appears in microsatellites. This is referred to as the stutter band and its identification is possible because they always follow the size of the main band. * Indicates polymorphic alleles, different from the expected band size.
(TIFF)

S2 Fig Principal component analysis (PCA) of 13 *Giardia duodenalis* isolates unambiguously assigned to A assemblage according to their multilocus genotypes based on tpi, gdh, and bg genes. Red denotes for AI assemblage isolate whereas blue indicates AIV assemblage samples.
(TIF)
