# Supplementary material for: Molecular genotyping, diversity studies and high-resolution molecular markers unveiled by microsatellites in Giardia duodenalis
Source: PLoS Negl Trop Dis. 2018 Nov 30;12(11):e0006928. doi: 10.1371/journal.pntd.0006928 (PMC6291164; doi:10.1371/journal.pntd.0006928)
Supplement: S1 Table — (DOCX) [file pntd.0006928.s001.docx]

Table S1. Designed primers, fragment size, SSR motif and position in *Giardia* assemblage A genome.

| **Primer ID** | **Primer F (5'-3')** | **Primer R (5'-3')** | **Fragment size (bp)** | **SSR motif** | **Contig ID** | **Start** | **End** |
| --- | --- | --- | --- | --- | --- | --- | --- |
| GduA01 | GGTTGATCGCTGGGAATAGA | TTTTCTGATGCGGCTAGGTT | 189 | (GA)5 | GLCHR02 | 1009368 | 1009556 |
| GED2 | CTTCCAACGCGAACTAGAGG | GCGAGTATCTGGAGGTGAGC | 141 | (GA)5 | GLCHR05 | 4297943 | 4298083 |
| GED3 | TTAGCGAGCTCCGGAATAGA | CCCGAGTCAGAGAGAGCAGT | 231 | (TC)5 | GLCHR01 | 455031 | 455261 |
| GduA02 | TGGAAGACGACAATCTGCTG | GCAATGGTGAGGGACATTTT | 168 | (CG)5 | GLCHR04 | 1240059 | 1240226 |
| GduA03 | AAGGTTCTCGACATGCATCC | CACGTTGAGCCATCCTCTTT | 195 | (GA)5 | GLCHR05 | 1565257 | 1565451 |
| GduA04 | TGGGTTCGGATTTTGTGTCT | GCGGCCATACTCTTGTTGAC | 187 | (CA)5 | GLCHR04 | 545016 | 545202 |
| GduA05 | TGGTCACAAATATCCCGTCA | TTCAACATGAATCAGGGCTTC | 231 | (TC)5 | GLCHR01 | 1457452 | 1457682 |
| GduA06 | GGCCAGGAACGTTCTTCTTA | GCACAAGGAAAGCTCCAAAG | 143 | (AG)5 | GLCHR05 | 4168808 | 4168950 |
| GED9 | AAGGTTCTCGACATGCATCC | CCAATCCTCTTGCTTTCCAC | 248 | (GA)5 | GLCHR05 | 1565204 | 1565451 |
| GED10 | TGGAAGACGACAATCTGCTG | GCAATGGTGAGGGACATTTT | 168 | (CG)5 | GLCHR04 | 1240059 | 1240226 |
| GduA07 | TGCCCTTGTCTATCGCTCTT | AGACATACCCGTTTCGCTTC | 224 | (AC)5 | GLCHR04 | 929274 | 929497 |
| GduA08 | CCAGAACGTGCAACTCGATA | TGCAATAGCGCCAGTAGAAA | 175 | (GT)5 | GLCHR04 | 919289 | 920126 |
| GduA09 | CATTGCAGTCAATCCGTCTTT | GGCACGACTGGGAAATCTTA | 180 | (CT)5 | GLCHR05 | 1237727 | 1237904 |
| GduA10 | GATAGCCATGCCATCAAAGG | CTCTACAGTCGCGGTCACCT | 232 | (GT)5 | GLCHR05 | 4328556 | 4328787 |
| GduA11 | TCAAGCACGCCTTCCTTATT | TTCCCCTGTCTGTCTCAAGG | 244 | (CT)5 | GLCHR05 | 2166264 | 2166507 |
| GduA12 | TCCGGTTTGCAAGCTTTTAC | AGCATCGCAGTTGTCCTTCT | 247 | (TG)5 | GLCHR05 | 2681542 | 2681788 |
| GduA13 | ACTGCGAGAGCAACGAGAGT | ACTGTGTGGGCAGATGATGA | 179 | (CA)5 | GLCHR03 | 401373 | 401551 |
| GGD8 | TGCACCAGTTGTTCCGATAA | TTCGCCGGTTAACTCTTCAT | 240 | (GC)5 | GLCHR04 | 2107881 | 2108127 |
| GduA14 | GCATGCAAATAAACGGAGGT | ATTCACAGGCAAGGATCCAC | 181 | (AC)5 | GLCHR02 | 448408 | 448609 |
| GduA15 | GACGGCTGCATCTCCTATTC | CCGGCGACAGACAACTATTTA | 140 | (CT)5 | GLCHR05 | 3412974 | 3413113 |
| GduA16 | GCTCCAGGCTTATCAACAGC | TCGGCTATGTTGTTCGTCTG | 243 | (CAA)6 | GLCHR05 | 3265764 | 3266006 |
| GduA17 | GCTTGCTTTTGCTTTGATCC | CGAACCTGTCCTCCCTCTCT | 205 | (CAG)7 | GLCHR05 | 1904801 | 1904181 |
| GduA18 | CGACGCTCTTGCTCTTCTTT | GCACCCAGTATTTCCCAATC | 242 | (GTT)9(GCT)5 | GLCHR04 | 2531922 | 2532563 |
| GduA19 | TGCCGCTTTCTGTAAACTCC | CGGACCTCTCTCGTTGACTT | 220 | (GCT)5 | GLCHR01 | 1308659 | 1308045 |
| GET5 | CGTGAGATCCTTGAATGCAC | ATAGAGGGTGGCAGGTGTGT | 238 | (CGA)5 | GLCHR05 | 2902690 | 2902826 |
| GET6 | AGGTCGTCTTTGCTGTGCTT | CGGAAACTATCGAACGAGGA | 230 | (CAT)5 | GLCHR05 | 1935458 | 1935687 |
| GduA20 | TCTTCCTCCTCCGACGACTA | GACTCCCCAGTCTCCTCCTC | 242 | (GAAGAG)3tat(GAA)4 | GLCHR03 | 1284936 | 1285568 |
| GET8 | TCGGCCTCTGGTCTTTACTG | ACGTTGGGGTACTCAACTGC | 207 | (TTG)4 | CH991808 | 199 | 810 |
| GduA21 | ATTCTTCCGGGGCTGTTACT | CTTTCCACGGCAGATAAACC | 194 | (TCG)4 | GLCHR04 | 515474 | 514863 |
| GduA22 | GCTACGACAGAGGCTCCAAC | CGTGCATTCTCATTGCTCAT | 194 | (GAC)4 | GLCHR01 | 703401 | 703594 |
| GduA23 | GTCCATCCGCTTGTGTTCTT | CTGGTGTGGCAGTCTCAAAA | 184 | (TGT)7 | GLCHR02 | 1142770 | 1142150 |
| GduA24 | CTCCCACGCGGAATACTAAG | GAGGCTTCTTTTGCATCCAG | 139 | (CAA)8 | GLCHR02 | 864558 | 863935 |
| GduA25 | CTCCGCCCTCCTATTCTTCT | AGCTTCTTACGGCATCCTGA | 198 | (CAA)10 | GLCHR05 | 3292744 | 3292115 |
| GGT4 | GGACATCGCCAAAAATTGAG | AGGCCCGCGAATATAAAACT | 220 | (ATC)5 | AACB02000075 | 10338 | 10557 |
| GduA26 | GAGGCGGTGCCAATACTACT | ACAAGGTGCAATCACGGACT | 248 | (GAT)5 | GLCHR01 | 483788 | 483174 |
| GGT6 | AGAACGAGAACGCAGCAAGT | GCGGACGCTTAACAATGTCT | 212 | (CAA)4 | GLCHR04 | 1369180 | 1369391 |
| GGT7 | CTCGTGAACCCACTGATTCC | GATTCCTCTGCTGGTGGTTC | 157 | (CCG)4 | GLCHR03 | 720331 | 720942 |
| GGT8 | TGTTACCTCTCCTGCCATCC | AACGCCAAGTCCAGACAAAC | 175 | (TCT)5 | GLCHR03 | 490377 | 490548 |
| GduA27 | ACTGGGGAGTCATTGGTCTG | ATACGTGCACGAACATTCCA | 176 | (CTG)4 | GLCHR04 | 158719 | 158108 |
| GduA28 | GCAAAGCATGGCCGTATC | GCCATGTCAGAGGCAGATG | 166 | (GCC)5 | GLCHR01 | 1173955 | 1173341 |

The reference genome for Contig ID and position in this table was the WB genome even though the primers were designed to anneal in both (WB and GS) genomes
