# Supplementary material for: Molecular genotyping, diversity studies and high-resolution molecular markers unveiled by microsatellites in Giardia duodenalis
Source: PLoS Negl Trop Dis. 2018 Nov 30;12(11):e0006928. doi: 10.1371/journal.pntd.0006928 (PMC6291164; doi:10.1371/journal.pntd.0006928)
Supplement: S2 Table — (DOCX) [file pntd.0006928.s002.docx]

Table S2. Designed primers, fragment size, SSR motif and position in *Giardia* GS genome.

| **Primer ID** | **Primer F (5'-3')** | **Primer R (5'-3')** | **Fragment size (bp)** | **SSR motif** | **Contig ID** | **Start** | **End** |
| --- | --- | --- | --- | --- | --- | --- | --- |
| B01 | ATCAACGGAACGGGTAGTTG | TCGGGGTCACGATTGATTTA | 227 | (CTG)6 | ACGJ01000950 | 8158 | 8775 |
| B02 | GTTACAGGGCCAGCTCTCTG | TTCCCCCATTCTTTCCTTCT | 248 | (CAG)6 | ACGJ01001563 | 31855 | 32472 |
| B03 | GCACATGGAACAGAGCTTGA | TCTTCTAGGTTTCCGCCTCA | 222 | (TCG)6 | ACGJ01002259 | 8890 | 9507 |
| B04 | CTTTTGTGCACGTGTTCCTG | CACACGGTGAGAGGTGCTTA | 106 | (TGC)6 | ACGJ01002301 | 20793 | 21410 |
| GduB01 | CCTCACAGAGGGCTAAGCAG | TCGGGCTATAGTCTCGTGCT | 218 | (TGA)9 | ACGJ01002662 | 21992 | 22618 |
| GduB02 | TCAACTGGCTGGTGAACTTG | CTATGCCTATCGCCTTCCAG | 165 | (ATTGCA)4 | ACGJ01002902 | 1059 | 1682 |
| B07 | CAACCACACGCTCAGAAAGA | ACCGTAGACATGGTGGAAGG | 187 | (AGC)6 | ACGJ01002929 | 30442 | 31059 |
| GduB03 | GCTCTAGCAGCTCTGCCACT | GCGAGGCTCTGCTATATTGG | 179 | (CAT)6 | ACGJ01002424 | 6899 | 7516 |
| GduB04 | CATCTATCGGCTGCCTGTTT | CTCCCAGGACTGCTTTCTGT | 224 | (ATA)4 | ACGJ01000230 | 11751 | 12362 |
| GduB05 | CGAGATGCTCAGTGACAAGC | CCTTAAACACGCACCAGTGA | 234 | (GAG)6 | ACGJ01002558 | 7426 | 8043 |
| GduB06 | GCTCCCCAACAAGTGCTTTA | GTGCGATATGGTTGCATCTG | 213 | (GCAGCC)3 | ACGJ01002471 | 9460 | 10077 |
| B12 | AATGGCCACTTGGAATGTGT | AAGCTGTTGACCCAGGTGAG | 208 | (AAGAA)3 | ACGJ01002439 | 1130 | 1744 |
| GduB07 | TAGAAAACCGGGTACGCAAC | GCACACATCTGCCTTTGCTA | 236 | (AAAGA)3 | ACGJ01002401 | 214 | 828 |
| GduB08 | TCGACCCCAATTGTTGAAAT | TTTGGTGAAGATGCTTGTGC | 247 | (ATTTA)3 | ACGJ01002394 | 1150 | 1764 |
| B15 | ATCGAGCACGCTTAACTTGG | TACCCCAGTCACATGCAGAA | 152 | (GAGCTG)3 | ACGJ01002375 | 1279 | 1896 |
| GduB09 | CCTGCTCAGGTCTCAAACAA | GCTTGTGACGCTCAAATGTC | 250 | (TTCTCA)3 | ACGJ01002319 | 31527 | 32144 |
| B17 | TGAGGGCGTTCGATAATAGG | AGCAAAGAGAGCAGGAGCAG | 223 | (TGT)6 | ACGJ01002305 | 9533 | 10150 |
| GduB10 | GGCTTGGATATTTGGTGTGC | GCCGGTCTAAGAGCCTACCT | 250 | (TGG)6 | ACGJ01002218 | 25344 | 25961 |
| B19 | TATATGGGGGTATGGGGGTA | ATTCAATTGGCTCCAGAAGG | 243 | (GGCGG)3 | ACGJ01002211 | 3048 | 3662 |
| GduB11 | AAACGATGCACTGCCTTTTC | TGGCAACGCAAAACTACAAG | 202 | (TGAA)3 | ACGJ01001296 | 52259 | 52870 |
