# Supplementary material for: Molecular genotyping, diversity studies and high-resolution molecular markers unveiled by microsatellites in Giardia duodenalis
Source: PLoS Negl Trop Dis. 2018 Nov 30;12(11):e0006928. doi: 10.1371/journal.pntd.0006928 (PMC6291164; doi:10.1371/journal.pntd.0006928)
Supplement: S3 Table — (DOCX) [file pntd.0006928.s003.docx]

Table S3. Designed primers, fragment size, SSR motif and position in *Giardia* assemblage E genome.

| **Primer ID** | **Primer F (5'-3')** | **Primer R (5'-3')** | **Fragment size (bp)** | **SSR motif** | **Contig ID** | **Start** | **End** |
| --- | --- | --- | --- | --- | --- | --- | --- |
| E001 | CCGTTCAAATGCTCCTCAAC | CGCTGCAATCCAATCAATTA | 246 | (GTA)4 | ACVC01000225 | 5795 | 6406 |
| E002 | AACGCCTTGGATATCACCAC | GCCAAACATGTGCCAATACA | 200 | (CTG)5 | ACVC01000111 | 35245 | 34631 |
| E003 | TGCAGCACCACCACTAGTTC | GGCTGCGCATTACGATAAGT | 199 | (CAGCAC)4 | ACVC01000109 | 30738 | 31361 |
| E004 | ACTACGCTTGACAGGCACAA | TCCGTTCCAAATACCGCTAC | 238 | (GTA)4 | ACVC01000103 | 6753 | 7364 |
| E005 | TTGGTAGCTGGCGTGTAATG | AGGAACGACAGGCTCACACT | 223 | (TCA)6 | ACVC01000158 | 74920 | 75537 |
| E006 | TCTCCTCAGCTTGTGGGTCT | CCCTCAGTGACGAGGCTCTA | 162 | (TGA)5 | ACVC01000108 | 27448 | 26834 |
| E007 | AGAGCACTGGCTGTTGGACT | GTGCGGATCTTTCCATTGAT | 246 | (TGC)5tgt(TGC)4 | ACVC01000109 | 49362 | 49991 |
| E008 | ACGTCTTCCTCCTCCGATG | CCCACAGCTGGCATTTACTC | 341 | (GAG)4tct(GAAGAG)3 | ACVC01000104 | 61790 | 62422 |
| E009 | TTCACCGCCAACTGTTACAA | GCGGTTCTTCAGCAGTTCTT | 290 | (ATC)8 | ACVC01000099 | 159568 | 158945 |
| E010 | GCAGTACTCGCCTTCAGTCC | TATGAGGAGGTCCGCACAAA | 281 | (ATG)11 | ACVC01000105 | 104386 | 105018 |
| E011 | ATGAAGGAGCTGCAAAGCAT | CCATCAGTCGCAACATCATT | 207 | (TG)10 | ACVC01000105 | 144112 | 144731 |
| E012 | AAGGTCCTGCTTGCACTTGT | ATTCACCGACCTCAATCACC | 167 | (TG)6 | ACVC01000112 | 33678 | 33067 |
| E013 | GAAGATCCTCGTGCCTGAAC | TTACATGCGCAGAATGTGGT | 332 | (ACGAA)3 | ACVC01000222 | 17420 | 18034 |
| E014 | TCAATCGAGCAGACGTTGAC | GGGCTGTCTATGCAGCAAAC | 269 | (TGTT)3 | ACVC01000222 | 27858 | 28469 |
| E015 | CTCGCGGACTTTTAGATTGC | GCACCTGGCTAGCACCTATC | 289 | (AATTGC)3 | ACVC01000123 | 12571 | 13188 |
| E016 | AAATCGCATCCTGAAAATCG | CCAGGTCCTAATTGGCTGAG | 206 | (CTC)5 | ACVC01000096 | 47982 | 47368 |
| E017 | CAACGGTCAGCTCAAGCATA | TGCGAGTAACTCCTGTTCCA | 288 | (GCAG)3 | ACVC01000215 | 84111 | 83500 |
| E018 | CAAGCAAAGATCTGGCAACA | ACCTCCATACCGTCGATCAG | 197 | (AAT)11 | ACVC01000421 | 2735 | 3367 |
| E019 | TAGGCTCAAGCAGCACACTG | ATCCGGTGGTATCAGCTCAG | 283 | (AGA)5 | ACVC01000195 | 61677 | 62291 |
| E020 | GGAGTTTGCGCAATTAGGAG | GTGTCCCGTTTCGCTAACAT | 298 | (GAT)6 | ACVC01000189 | 13455 | 14072 |
