# Supplementary material for: Molecular genotyping, diversity studies and high-resolution molecular markers unveiled by microsatellites in Giardia duodenalis
Source: PLoS Negl Trop Dis. 2018 Nov 30;12(11):e0006928. doi: 10.1371/journal.pntd.0006928 (PMC6291164; doi:10.1371/journal.pntd.0006928)
Supplement: S4 Table — (DOCX) [file pntd.0006928.s004.docx]

Table S4. Designed primers, fragment size, SSR motif and position of *Giardia* primers shared by all assemblage genomes.

| **Primer ID** | **Primer F (5'-3')** | **Primer R (5'-3')** | **Fragment size (bp)** | **SSR motif** |
| --- | --- | --- | --- | --- |
| ABE01 | CACTTCTACGGTGAYGASAAG | ATCTTGTGYCCCGAGAG | 386 | (GAG)4 |
| ABE02 | AAAGGARCTGTAYATGAACGG | CCYTCCTGTTTGAYAGCCAT | 204 | (AGCA)3 |
| ABE03 | AGAAGRAGCTTGACACACTC | AGAACATGGTMTCRGAGGA | 325 | (GCA)4 |
| ABE04 | AAGAARTACACGTCTGATCC | GATAATTGAGTAAAGGAGCG | 335 | (TATG)3 |
| GduABE01 | GYGAGGAGGAGGTAGAGGT | GCTTRGGGATGAAGAGGAT | 333 | (TCT)4 |
| ABE06 | TGYGTGACCTCCATCTTCA | TCCAGTGYATGCTCTGYG | 253 | (GAG)4 |
| ABE07 | AAATTGTGCCTCAGTAGTGT | AAGCGGTAYACRGGGTAGAT | 291 | (TCA)4 |
| ABE08 | GCAGAGCTCTGYGTTGAAGG | ACCACCTCRCGAACAAGTGT | 107 | (GAA)4 |
| ABE09 | CCTTGGCTCTRCTRGCATA | TGGTCCTGGGRAGCTGYATC | 240 | (TTGC)3 |
| ABE10 | CATRCTGCCACGACGACT | GCCTGAYTATGCGTATTTGAGG | 328 | (GAT)4 |
| ABE11 | AAATCTGCCARGTARCC | ACACRCAGAGCATGATAAARTC | 268 | (TCT)4 |
| ABE12 | GACCGCCGCGTCATGCG | GCCTGGCGYTCACGGTTGTAGC | 299 | (GCT)4 |
| ABE13 | AGAGGGATTCTCCTGCTRAG | CTGGCAGARGATGGRAGC | 233 | (ATC)4 |
| ABE14 | CCCGCAGRTGCTCYTCACG | CGRCAGCTYGCAGTTAGGCT | 283 | (ATG)4 |
| ABE15 | TGCTTTGCAATCTCRTCTGC | GARCAGCATCGCCGYCTCA | 236 | (CTG)4 |
| GduABE02 | TGCATCGYTGCTCTGCCTTCT | CCACWGCGGTCAYGGTAAGC | 290 | (AGA)4 |
| ABE17 | GGRAACGTGATGAGYCAAGAG | GCACGMGAAATRGCATAG | 329 | (CTTTA)3 |
| ABE18 | AGCRTGCAGGGARTAYCTC | ATGCACTCGTCGATMCG | 237 | (GAT)4 |
| ABE19 | ACGGATACATAAAGAAYGARCG | ACTGCAAABCCAAAGTCWG | 214 | (CTT)4 |
| ABE20 | GGCGARAGYTGAGTGGC | GTGCCCTYGCRATCCGTAC | 271 | (CGC)4 |
| ABE21 | GATATGGGCGAAGGYTTTG | CGACYTCTGTGACRTTCTCTT | 295 | (ATAG)3 |
| ABE22 | ACAATGAAACGGGRCCA | CCATTRTGCTCYGCCAT | 211 | (TTGG)3 |
| ABE23 | ACCACCTGCTGYTTYCCG | AAGACGTACTGCGAAGARTGG | 281 | (GGC)4 |
| ABE24 | ATGGAGCCGTTCTAYCTTGTT | CTCCTTCATGCCYTTRCG | 318 | (GCAG)3 |
| GduABE03 | AGCTTGCCAAGTGGAGGAG | AAGAGCTCGATGCTRAACGG | 349 | (CGT)4 |
| ABE26 | GTGTACTTGAGYGGAAAYGAAG | ATCGARGAGGTCTCYGTCAC | 326 | (CTG)4 |
| ABE27 | GCCTCCGCCCCGTGAAG | ACGACGCTGAACTGCCGCT | 259 | (GGCG)3 |
| ABE28 | CAGRTGGATAAAGGARAAGGAG | ACAACTGCRTTYAGAAGCTG | 363 | (CGT)4 |
| ABE29 | ACAGTAAAGYCTCGTCCTTGTG | GTCTGCGTCYRCTGCYCC | 200 | (CGA)4 |
| ABE30 | GCACCAGCTACKACRAACAGAAT | GTGTGCACYAAGATGGAGCC | 344 | (CAAG)3 |
| GduABE04 | TGATCTCTGTCTCCTTGATKGG | CATGCAGGCTGARAGTGCTC | 182 | (TCC)4 |
| ABE32 | CTGCWTACGGGYTTTTC | GCRTTGACCTTTCRCTTCTT | 283 | (ACAA)3 |
| ABE33 | CGTGTRAGCTTCTCTATC | TAYGGHATGGTTGAGAATAT | 356 | (AAG)4 |
| ABE34 | CCATCTGCCATTCTRAAAAGAG | GCAAACTCAGCRAAGTCTA | 236 | (TCAG)3 |
| ABE35 | CTGARAATGTGGARAACTGC | AATATACGCTTGAGATAAAGRTG | 346 | (TGTC)3 |
| ABE36 | TTGTCTGCTGGGAACTCA | GACCTWCCTGCCATAGACT | 206 | (CATA)3 |
