# Supplementary material for: Molecular genotyping, diversity studies and high-resolution molecular markers unveiled by microsatellites in Giardia duodenalis
Source: PLoS Negl Trop Dis. 2018 Nov 30;12(11):e0006928. doi: 10.1371/journal.pntd.0006928 (PMC6291164; doi:10.1371/journal.pntd.0006928)
Supplement: S5 Table — (DOCX) [file pntd.0006928.s005.docx]

Table 5S. Specimens included in the present study according to their genotype and source.

| **Number of isolates** | **Isolate** | **Genotype** | **Source** |
| --- | --- | --- | --- |
| 1 | HC01 | AII | Hospital |
| 2 | HC06 | AII | Hospital |
| 3 | HC10 | AII | Hospital |
| 4 | HC11 | AII | Hospital |
| 5 | HC18 | AII | Hospital |
| 6 | HC22 | AII | Hospital |
| 7 | HC27 | AII | Hospital |
| 8 | HC28 | AII | Hospital |
| 9 | HC29 | AII | Hospital |
| 10 | HC31 | AII | Hospital |
| 11 | HC35 | AII | Hospital |
| 12 | HC40 | AII | Hospital |
| 13 | HC42 | AII | Hospital |
| 14 | HC48 | AII | Hospital |
| 15 | HC50 | AII | Hospital |
| 16 | HC51 | AII | Hospital |
| 17 | DC12 | AII | Daycare |
| 18 | DC13 | AII | Daycare |
| 19 | DC15 | AII | Daycare |
| 20 | DC25 | AII | Daycare |
| 21 | DC27 | AII | Daycare |
| 22 | DC28 | AII | Daycare |
| 23 | DC03R | AI | Veterinary |
| 24 | HC14 | BIV | Hospital |
| 25 | HC15 | BIV | Hospital |
| 26 | HC17 | BIV | Hospital |
| 27 | HC20 | BIV | Hospital |
| 28 | HC24 | BIV | Hospital |
| 29 | HC25 | BIV | Hospital |
| 30 | HC30 | BIV | Hospital |
| 31 | HC32 | BIV | Hospital |
| 32 | HC33 | BIII | Hospital |
| 33 | HC34 | BIV | Hospital |
| 34 | HC38 | BIV | Hospital |
| 35 | HC41 | BIV | Hospital |
| 36 | HC43 | BIV | Hospital |
| 37 | HC45 | BIV | Hospital |
| 38 | HC47 | BIV | Hospital |
| 39 | DC04 | BIII | Daycare |
| 40 | DC09 | BIII | Daycare |
| 41 | DC10 | BIII | Daycare |
| 42 | DC16 | BIV | Daycare |
| 43 | DC17 | BIV | Daycare |
| 44 | DC18 | BIII | Daycare |
| 45 | DC21 | BIV | Daycare |
| 46 | DC24 | BIV | Daycare |
| 47 | D1R | C | Veterinary |
| 48 | D11R | C | Veterinary |
| 49 | D16R | C | Veterinary |
| 50 | D19R | C | Veterinary |
| 51 | ENV02 | C | Environmental |
| 52 | D2R | D | Veterinary |
| 53 | D12R | D | Veterinary |
| 54 | D30R | D | Veterinary |
| 55 | D31R | D | Veterinary |
| 56 | D32R | D | Veterinary |
| 57 | VETL | E | Veterinary |
