# Supplementary material for: Molecular genotyping, diversity studies and high-resolution molecular markers unveiled by microsatellites in Giardia duodenalis
Source: PLoS Negl Trop Dis. 2018 Nov 30;12(11):e0006928. doi: 10.1371/journal.pntd.0006928 (PMC6291164; doi:10.1371/journal.pntd.0006928)
Supplement: S6 Table — (DOCX) [file pntd.0006928.s006.docx]

Table S6. Gene ontology classification of *G. duodenalis* genes regarding GO category of Biological process.

| **Protein sequences (Biological processes)** | **Number of GO terms** |
| --- | --- |
| immune system process | 37 |
| reprodutive process | 41 |
| locomotion | 42 |
| reproduction | 45 |
| multi-organism process | 47 |
| negative regulation of biological process | 70 |
| positive regulation of biological process | 76 |
| signaling | 83 |
| localization | 107 |
| response to stimulus | 134 |
| multicellular organismal process | 138 |
| regulation of biological process | 138 |
| cellular process | 245 |
| single-organism process | 233 |
| metabolic process | 180 |
| biological regulation | 146 |
| cellular component organization or biogenesis | 143 |
| developmental process | 142 |
