# Supplementary material for: Molecular genotyping, diversity studies and high-resolution molecular markers unveiled by microsatellites in Giardia duodenalis
Source: PLoS Negl Trop Dis. 2018 Nov 30;12(11):e0006928. doi: 10.1371/journal.pntd.0006928 (PMC6291164; doi:10.1371/journal.pntd.0006928)
Supplement: S7 Table — (DOCX) [file pntd.0006928.s007.docx]

Table S7. Gene ontology classification of *G. duodenalis* genes regarding GO category of Cellular components.

| **Protein sequences (Cellular components)** | **Number of GO terms** |
| --- | --- |
| cell projection part | 42 |
| intrinsic component of membrane | 42 |
| endomembrane system | 47 |
| cilium | 51 |
| organelle lumen | 61 |
| cell projection | 77 |
| plasma membrane | 88 |
| protein complex | 90 |
| cell periphery | 94 |
| non-membrane-bounded organelle | 145 |
| intracellular organelle part | 147 |
| intracellular part | 256 |
| intracellular | 256 |
| intracellular organelle | 244 |
| membrane-bounded organelle | 198 |
