# Supplementary material for: Molecular genotyping, diversity studies and high-resolution molecular markers unveiled by microsatellites in Giardia duodenalis
Source: PLoS Negl Trop Dis. 2018 Nov 30;12(11):e0006928. doi: 10.1371/journal.pntd.0006928 (PMC6291164; doi:10.1371/journal.pntd.0006928)
Supplement: S8 Table — (DOCX) [file pntd.0006928.s008.docx]

Table S8. Gene ontology classification of *G. duodenalis* genes regarding GO category of Molecular function.

| **Protein sequences (Molecular function)** | **Number of GOterms** |
| --- | --- |
| macromolecular complex binding | 29 |
| carbohydrate derivative binding | 46 |
| transferase activity | 61 |
| small molecule binding | 61 |
| ion binding | 84 |
| hydrolase activity | 104 |
| protein binding | 145 |
| organic cyclic compound binding | 105 |
| heterocyclic compound binding | 105 |
