# Supplementary material for: Molecular genotyping, diversity studies and high-resolution molecular markers unveiled by microsatellites in Giardia duodenalis
Source: PLoS Negl Trop Dis. 2018 Nov 30;12(11):e0006928. doi: 10.1371/journal.pntd.0006928 (PMC6291164; doi:10.1371/journal.pntd.0006928)
Supplement: S9 Table — (DOCX) [file pntd.0006928.s009.docx]

Table S9. Amplification results and proteins associated with SSR loci in genetic assemblage A.

| **SSR name** | **Specific amplification** | **Final results** | **Polymorphic** | **Protein name** |
| --- | --- | --- | --- | --- |
| GduA01 | yes | suitable | 0.1574 | XP_001709707.1Hypothetical protein GL50803_28337 [Giardia lamblia ATCC 50803] |
| GED2 | no | unsuitable | - | XP_001705829.1VSP [Giardia lamblia ATCC 50803] |
| GED3 | yes | unsuitable | - | XP_001705121.1Protein 21.1 [Giardia lamblia ATCC 50803] |
| GduA02 | yes | suitable | 0.1167 | ---NA--- |
| GduA03 | yes | suitable | monomorphic | XP_001709265.1Hypothetical protein GL50803_137718 [Giardia lamblia ATCC 50803] |
| GduA04 | yes | suitable | monomorphic | EFD95044.1hypothetical protein GL50803_115571 [Giardia lamblia ATCC 50803] |
| GduA05 | yes | suitable | monomorphic | ESU35046.1Serine/threonine protein kinase [Giardia intestinalis] |
| GduA06 | yes | suitable | monomorphic | ESU37109.1Chromosome segregation protein SMC [Giardia intestinalis] |
| GED9 | no | unsuitable | - | XP_001708193.1Protein 21.1 [Giardia lamblia ATCC 50803] |
| GED10 | yes | unsuitable | - | XP_001707457.1Hypothetical protein GL50803_4439 [Giardia lamblia ATCC 50803] |
| GduA07 | yes | suitable | monomorphic | XP_001708765.1Hypothetical protein GL50803_16599 [Giardia lamblia ATCC 50803] |
| GduA08 | yes | suitable | 0.0797 | XP_001708514.1Coiled-coil protein [Giardia lamblia ATCC 50803] |
| GduA09 | yes | suitable | monomorphic | ESU38794.1Hypothetical protein DHA2_8854 [Giardia intestinalis] |
| GduA10 | yes | suitable | 0.3724 | XP_001706599.1Hypothetical protein GL50803_29308 [Giardia lamblia ATCC 50803] |
| GduA11 | yes | suitable | monomorphic | ESU39628.1Putative WD-repeat family protein [Giardia intestinalis] |
| GduA12 | yes | suitable | monomorphic | EFD94933.1hypothetical protein GL50803_17123 [Giardia lamblia ATCC 50803] |
| GduA13 | yes | suitable | monomorphic | ESU39032.1Putative AAA domain protein [Giardia intestinalis] |
| GGD8 | yes | unsuitable | - | XP_001708589.1H-SHIPPO 1 [Giardia lamblia ATCC 50803] |
| GduA14 | yes | suitable | monomorphic | ESU39628.1Putative WD-repeat family protein [Giardia intestinalis] |
| GduA15 | yes | suitable | monomorphic | XP_001706599.1Hypothetical protein GL50803_29308 [Giardia lamblia ATCC 50803] |
| GduA16 | yes | suitable | 0.3679 | ---NA--- |
| GduA17 | yes | suitable | 0.083 | XP_001709401.1Hypothetical protein GL50803_3341 [Giardia lamblia ATCC 50803] |
| GduA18 | yes | suitable | monomorphic | XP_001704576.1Kinase, NEK [Giardia lamblia ATCC 50803] |
| GduA19 | yes | suitable | 0.0797 | ESU38956.1Hypothetical protein DHA2_152326 [Giardia intestinalis] |
| GET5 | no | unsuitable | - | XP_001708799.1VSP [Giardia lamblia ATCC 50803] |
| GET6 | no | unsuitable | - | XP_001709378.1Protein required for cell viability [Giardia lamblia ATCC 50803] |
| GduA20 | yes | suitable | 0.1574 | ---NA--- |
| GET8 | no | unsuitable | - | XP_001705784.1VSP [Giardia lamblia ATCC 50803] |
| GduA21 | yes | suitable | 0.2468 | AAN52114.1variant-specific surface protein AS11 [Giardia intestinalis] |
| GduA22 | yes | suitable | monomorphic | XP_001706499.1Hypothetical protein GL50803_17332 [Giardia lamblia ATCC 50803] |
| GduA23 | yes | suitable | monomorphic | ESU37629.1Hypothetical protein DHA2_152676 [Giardia intestinalis] |
| GduA24 | yes | suitable | 0.5388 | XP_001706211.1Kinase [Giardia lamblia ATCC 50803] |
| GduA25 | yes | suitable | 0.0865 | EFD95319.1hypothetical protein GL50803_114751 [Giardia lamblia ATCC 50803] |
| GGT4 | no | unsuitable | - | ESU34706.1Hypothetical protein DHA2_150876 [Giardia intestinalis] |
| GduA26 | yes | suitable | 0.141 | XP_001707868.1Hypothetical protein GL50803_5458 [Giardia lamblia ATCC 50803] |
| GGT6 | yes | unsuitable | - | XP_001706056.1Hypothetical protein GL50803_17008 [Giardia lamblia ATCC 50803] |
| GGT7 | yes | unsuitable | - | ESU35918.1EGF family protein [Giardia intestinalis] |
| GGT8 | yes | unsuitable | - | XP_001706278.1Hypothetical protein GL50803_6120 [Giardia lamblia ATCC 50803] |
| GduA27 | yes | suitable | monomorphic | XP_001707268.1Protein 21.1 [Giardia lamblia ATCC 50803] |
| GduA28 | yes | suitable | monomorphic | XP_001709173.1Hypothetical protein GL50803_37173 [Giardia lamblia ATCC 50803] |
