# Supplementary material for: Molecular genotyping, diversity studies and high-resolution molecular markers unveiled by microsatellites in Giardia duodenalis
Source: PLoS Negl Trop Dis. 2018 Nov 30;12(11):e0006928. doi: 10.1371/journal.pntd.0006928 (PMC6291164; doi:10.1371/journal.pntd.0006928)
Supplement: S10 Table — (DOCX) [file pntd.0006928.s010.docx]

Table S10. Amplification results and proteins associated with SSR loci in genetic assemblage B.

| **SSR name** | **Specific amplification** | **Final results** | **Polymorphic** | **Protein name** |
| --- | --- | --- | --- | --- |
| B01 | yes | unsuitable | - | EET01952.1DGTP triphosphohydrolase [Giardia intestinalis ATCC 50581] |
| B02 | yes | unsuitable | - | ESU42530.1Hypothetical protein GSB_151269 [Giardia intestinalis] |
| B03 | no | unsuitable | - | ESU43700.1Variant-specific surface protein [Giardia intestinalis] |
| B04 | yes | unsuitable | - | ---NA--- |
| GduB01 | yes | Suitable | 0.3962 | EES99569.1Hypothetical protein GL50581_3205 [Giardia intestinalis ATCC 50581] |
| GduB02 | yes | suitable | 0.5108 | ESU45262.1Hypothetical protein GSB_150744, partial [Giardia intestinalis] |
| B07 | yes | unsuitable | - | ESU44666.1Ankyrin repeat protein, partial [Giardia intestinalis] |
| GduB03 | yes | suitable | 0.1899 | ESU42613.1Protein Translation Initiation Factor 1 (IF-1) (or SUI1) [Giardia intestinalis] |
| GduB04 | yes | suitable | monomorphic | KWX13557.1hypothetical protein QR46_2429 [Giardia intestinalis assemblage B] |
| GduB05 | yes | suitable | 0.3257 | ---NA--- |
| GduB06 | yes | suitable | 0.4342 | ESU43420.1Serine/threonine protein kinase [Giardia intestinalis] |
| B12 | yes | unsuitable | - | ESU45136.1Ankyrin repeat protein [Giardia intestinalis] |
| GduB07 | yes | suitable | monomorphic | KWX12665.1hypothetical protein QR46_3332 [Giardia intestinalis assemblage B] |
| GduB08 | yes | suitable | monomorphic | XP_001705159.1GTP-binding nuclear protein RAN/TC4 [Giardia lamblia ATCC 50803] |
| B15 | yes | unsuitable | - | ESU40807.1Ankyrin repeat protein, partial [Giardia intestinalis] |
| GduB09 | yes | suitable | 0.2078 | EET00294.1Hypothetical protein GL50581_2482 [Giardia intestinalis ATCC 50581] |
| B17 | yes | unsuitable | - | EET00387.1Hypothetical protein GL50581_2366 [Giardia intestinalis ATCC 50581] |
| GduB10 | yes | suitable | 0.201 | ESU41568.1hypothetical protein GSB_151643 [Giardia intestinalis] |
| B19 | no | unsuitable | - | ---NA--- |
| GduB11 | yes | suitable | monomorphic | EET01741.1Hypothetical protein GL50581_1011 [Giardia intestinalis ATCC 50581] |
