# Supplementary material for: Molecular genotyping, diversity studies and high-resolution molecular markers unveiled by microsatellites in Giardia duodenalis
Source: PLoS Negl Trop Dis. 2018 Nov 30;12(11):e0006928. doi: 10.1371/journal.pntd.0006928 (PMC6291164; doi:10.1371/journal.pntd.0006928)
Supplement: S11 Table — (DOCX) [file pntd.0006928.s011.docx]

Table S11. Amplification results and proteins associated with SSR loci in genetic assemblage E.

| **SSR Name** | **Specific Amplification** | **Protein Name** |
| --- | --- | --- |
| E01 | yes | EFO61499.1Hypothetical protein GLP15_4966 [Giardia lamblia P15] |
| E02 | no | ---NA--- |
| E03 | yes | ---NA--- |
| E04 | yes | ---NA--- |
| E05 | yes | EFO62925.1Hypothetical protein GLP15_322 [Giardia lamblia P15] |
| E06 | yes | ---NA--- |
| E07 | yes | EFO63980.1Hypothetical protein GLP15_36 [Giardia lamblia P15] |
| E08 | yes | ---NA--- |
| E09 | yes | EFO64331.1Hypothetical protein GLP15_1207 [Giardia lamblia P15] |
| E10 | yes | EFO64086.1Hypothetical protein GLP15_3472 [Giardia lamblia P15] |
| E11 | yes | EFO64108.1Hypothetical protein GLP15_3496 [Giardia lamblia P15] |
| E12 | yes | ---NA--- |
| E13 | yes | ESU39186.1Kinesin motor domain protein [Giardia intestinalis] |
| E14 | yes | ---NA--- |
| E15 | yes | EFO63651.1Protein 21.1 [Giardia lamblia P15] |
| E16 | yes | EFO64407.1Hypothetical protein GLP15_929 [Giardia lamblia P15] |
| E17 | yes | ---NA--- |
| E18 | no | EFO61049.1VSP [Giardia lamblia P15] |
| E19 | no | EFO62195.1Hypothetical protein GLP15_4624 [Giardia lamblia P15] |
| E20 | yes |  |
