# Supplementary material for: Molecular genotyping, diversity studies and high-resolution molecular markers unveiled by microsatellites in Giardia duodenalis
Source: PLoS Negl Trop Dis. 2018 Nov 30;12(11):e0006928. doi: 10.1371/journal.pntd.0006928 (PMC6291164; doi:10.1371/journal.pntd.0006928)
Supplement: S12 Table — (DOCX) [file pntd.0006928.s012.docx]

Table S12. Amplification results and proteins associated with shared SSR loci.

| **SSR name** | **Specific amplification** | **Final results** | **Polymorphic** | **Protein name** |
| --- | --- | --- | --- | --- |
| ABE01 | no | unsuitable | - | ESU38083.1Cysteine desulfurase / Selenocysteine lyase [Giardia intestinalis] |
| ABE02 | no | unsuitable | - | ESU36802.1Dynein beta chain, ciliary [Giardia intestinalis] |
| ABE03 | no | unsuitable | - | ESU36815.1Ankyrin repeat protein [Giardia intestinalis] |
| ABE04 | no | unsuitable | - | XP_001706318.1Dynein heavy chain [Giardia lamblia ATCC 50803] |
| GduABE01 | no | suitable | 0.0415 | BAD83616.1cytosolic-type hsp90, partial [Giardia intestinalis] |
| ABE06 | no | unsuitable | - | ---NA--- |
| ABE07 | no | unsuitable | - | XP_001707895.1Copine I [Giardia lamblia ATCC 50803] |
| ABE08 | no | unsuitable | - | ESU36531.1Axoneme central apparatus protein [Giardia intestinalis] |
| ABE09 | no | unsuitable | - | ESU42557.1Kinesin motor domain protein [Giardia intestinalis] |
| ABE10 | no | unsuitable | - | XP_001704140.1Kinase, CK1 Casein kinase [Giardia lamblia ATCC 50803] |
| ABE11 | no | unsuitable | - | ---NA--- |
| ABE12 | no | unsuitable | - | XP_001707409.1Median body protein [Giardia lamblia ATCC 50803] |
| ABE13 | no | unsuitable | - | ---NA--- |
| ABE14 | no | unsuitable | - | ESU39610.1U3 small nucleolar ribonucleoprotein IMP4 [Giardia intestinalis] |
| ABE15 | no | unsuitable | - | XP_001706431.1Dynein heavy chain [Giardia lamblia ATCC 50803] |
| GduABE02 | yes | suitable | Monomorphic | XP_001707051.1Hypothetical protein GL50803_19294 [Giardia lamblia ATCC 50803] |
| ABE17 | no | unsuitable | - | ESU39516.1Putative TPR repeat family protein [Giardia intestinalis] |
| ABE18 | no | unsuitable | - | ---NA--- |
| ABE19 | no | unsuitable | - | ---NA--- |
| ABE20 | no | unsuitable | - | XP_001703936.1Kinase, NEK [Giardia lamblia ATCC 50803] |
| ABE21 | no | unsuitable | - | XP_001705832.1Hypothetical protein GL50803_94542 [Giardia lamblia ATCC 50803] |
| ABE22 | no | unsuitable | - | KWX14050.1Glucosyltransferase [Giardia intestinalis assemblage B] |
| ABE23 | no | unsuitable | - | XP_001706431.1Dynein heavy chain [Giardia lamblia ATCC 50803] |
| ABE24 | no | unsuitable | - | ---NA--- |
| GduABE03 | yes | suitable | monomorphic | ---NA--- |
| ABE26 | no | unsuitable | - | XP_001707389.1Dynein heavy chain [Giardia lamblia ATCC 50803] |
| ABE27 | no | unsuitable | - | XP_001705437.1Hypothetical protein GL50803_17351 [Giardia lamblia ATCC 50803] |
| ABE28 | no | unsuitable | - | XP_001704713.1Dynein heavy chain, putative [Giardia lamblia ATCC 50803] |
| ABE29 | no | unsuitable | - | ---NA--- |
| ABE30 | no | unsuitable | - | ESU35077.1Dynein heavy chain [Giardia intestinalis] |
| GduABE04 | yes | suitable | monomorphic | ---NA--- |
| ABE32 | no | unsuitable | - | ESU38797.1DNA polymerase delta catalytic subunit [Giardia intestinalis] |
| ABE33 | no | unsuitable | - | XP_001708270.1Nuclear ATP/GTP-binding protein [Giardia lamblia ATCC 50803] |
| ABE34 | no | unsuitable | - | XP_001708157.1Hypothetical protein GL50803_7405 [Giardia lamblia ATCC 50803] |
| ABE35 | no | unsuitable | - | ---NA--- |
| ABE36 | no | unsuitable | - | ESU38894.1ATP-dependent RNA helicase [Giardia intestinalis] |
