# Supplementary material for: Molecular genotyping, diversity studies and high-resolution molecular markers unveiled by microsatellites in Giardia duodenalis
Source: PLoS Negl Trop Dis. 2018 Nov 30;12(11):e0006928. doi: 10.1371/journal.pntd.0006928 (PMC6291164; doi:10.1371/journal.pntd.0006928)
Supplement: S14 Table — This table presents the results of each SSR loci in genetic assemblage B that were considered suitable for genotyping analysis. HE means expected heterozygosity Ho means observed heterozygosity PIC means polymorphic information content. The allelic variation represents the length variation of the alleles in the population. The number of alleles represents the number of alleles in the population. (DOCX) [file pntd.0006928.s014.docx]

Table S14. Characterization of SSR loci in genetic assemblage B.

| Locus | Number of Alleles | Allelic variation (bp) | H_E_ | H_O_ | PIC |
| --- | --- | --- | --- | --- | --- |
| GduB01 | 5 | 209-230 | 0.4508 | 0.1111 | 0.3962 |
| GduB02 | 3 | 153-165 | 0.591 | 0.1 | 0.5108 |
| GduB03 | 3 | 173-185 | 0.2079 | 0.1111 | 0.1899 |
| GduB04 | 1 | 224 | 0 | 0 | 0 |
| GduB05 | 3 | 216-234 | 0.3763 | 0 | 0.3257 |
| GduB06 | 5 | 188-224 | 0.4705 | 0.0435 | 0.4342 |
| GduB07 | 1 | 236 | 0 | 0 | 0 |
| GduB08 | 1 | 247 | 0 | 0 | 0 |
| GduB09 | 2 | 250-253 | 0.241 | 0 | 0.2078 |
| GduB10 | 2 | 244-250 | 0.2319 | 0 | 0.201 |
| GduB11 | 1 | 202 | 0 | 0 | 0 |
