# Supplementary material for: Molecular genotyping, diversity studies and high-resolution molecular markers unveiled by microsatellites in Giardia duodenalis
Source: PLoS Negl Trop Dis. 2018 Nov 30;12(11):e0006928. doi: 10.1371/journal.pntd.0006928 (PMC6291164; doi:10.1371/journal.pntd.0006928)
Supplement: S16 Table — (DOCX) [file pntd.0006928.s016.docx]

**List of accession numbers/ID numbers for proteins mentioned in the text**

VS41_GIAIN ame: Full=Variant-specific surface VSP4A1 ame: Full=CRISP-90 Flags: Precursor: **Uniprot number P92127**

ANR35_HUMAN ame: Full=Ankyrin repeat domain-containing 35U: **Uniprot number Q8N283**

CST1_CAEEL ame: Full=Serine threonine- kinase cst-1 ame: Full=STE20-like kinase 1 ame: Full=STE20-like kinase MST ame: Full=cMST Contains: ame: Full=Serine threonine- kinase cst-1 37kDa subunit Contains: ame: Full=Serine threonine- kinase cst-1 18kDa subunit: **Uniprot number Q9NB31**

NFS1_DROME ame: Full=Probable cysteine desulfurase, mitochondrial Flags: Precursor: **Uniprot number Q9VKD3**

Triosephosphate isomerase: **Uniprot number P36186**

Beta giardin: **Uniprot number Q6QDY1**

Glutamate dehidrogenase: **Uniprot number M9TFM4**
