# Supplementary figures and images for: Molecular genotyping, diversity studies and high-resolution molecular markers unveiled by microsatellites in Giardia duodenalis
Source: PLoS Negl Trop Dis. 2018 Nov 30;12(11):e0006928. doi: 10.1371/journal.pntd.0006928 (PMC6291164; doi:10.1371/journal.pntd.0006928)

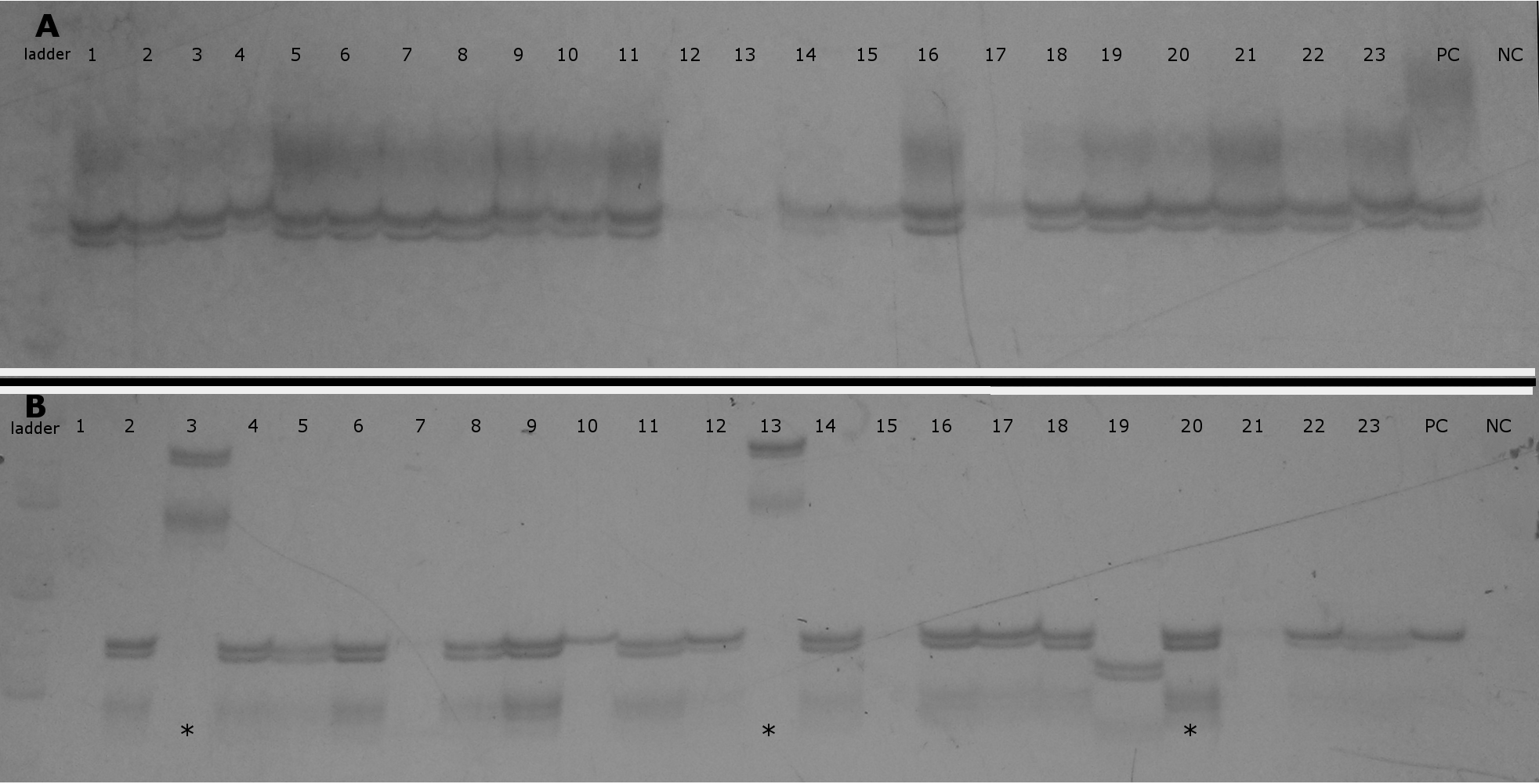

Supplement: S1 Fig — A—PCR products for the monomorphic marker GduA17 (205bp). B–PCR products for the polymorphic marker GduA20 (242bp) which three different alleles could be identified in the specimens from the same genetic assemblage. The numbering follows the number of the samples in the S5 Table. PC means positive control. NC means negative control. Double bands present in the same individual in this gel are a PCR amplification artifact that normally appears in microsatellites. This is referred to as the stutter band and its identification is possible because they always follow the size of the main band. * Indicates polymorphic alleles, different from the expected band size. (TIFF) [file pntd.0006928.s017.TIFF]

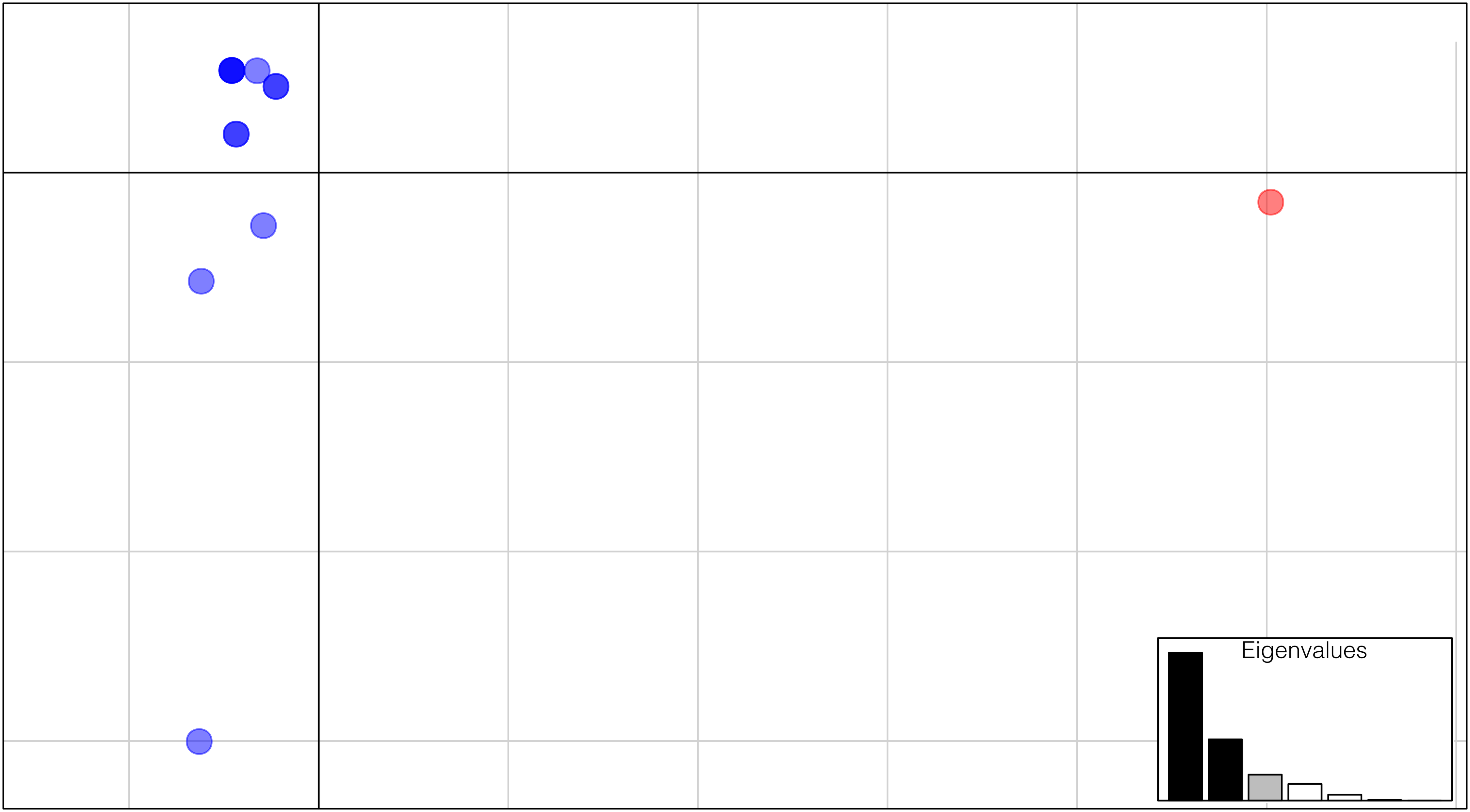

Supplement: S2 Fig — Red denotes for AI assemblage isolate whereas blue indicates AIV assemblage samples. (TIF) [file pntd.0006928.s018.tif]
